# Fluidity and Lipid Composition of Membranes of Peroxisomes, Mitochondria and the ER From Oleic Acid-Induced *Saccharomyces cerevisiae*

**DOI:** 10.3389/fcell.2020.574363

**Published:** 2020-10-29

**Authors:** Katharina Reglinski, Laura Steinfort-Effelsberg, Erdinc Sezgin, Christian Klose, Harald W. Platta, Wolfgang Girzalsky, Christian Eggeling, Ralf Erdmann

**Affiliations:** ^1^Leibniz-Institute of Photonic Technologies, Jena, Germany; ^2^Institute of Applied Optics and Biophysics, Friedrich-Schiller University Jena, Jena, Germany; ^3^MRC Human Immunology Unit, Weatherall Institute of Molecular Medicine, University of Oxford, Oxford, United Kingdom; ^4^University Hospital Jena, Jena, Germany; ^5^Systems Biochemistry, Medical Faculty, Ruhr-University Bochum, Bochum, Germany; ^6^Science for Life Laboratory, Department of Women’s and Children’s Health, Karolinska Institutet, Solna, Sweden; ^7^Lipotype GmbH, Dresden, Germany; ^8^Biochemistry of Intracelluar Transport, Ruhr-University Bochum, Bochum, Germany; ^9^Jena Center for Soft Matter (JCSM), Jena, Germany

**Keywords:** peroxisomes, mitochondria, ER, lipidomics, peroxin

## Abstract

The maintenance of a fluid lipid bilayer is key for organelle function and cell viability. Given the critical role of lipid compositions in determining membrane properties and organelle identity, it is clear that cells must have elaborate mechanism for membrane maintenance during adaptive responses to environmental conditions. Emphasis of the presented study is on peroxisomes, oleic acid-inducible organelles that are essential for the growth of yeast under conditions of oleic acid as single carbon source. Here, we isolated peroxisomes, mitochondria and ER from oleic acid-induced *Saccharomyces cerevisiae* and determined the lipid composition of their membranes using shotgun lipidomics and compared it to lipid ordering using fluorescence microscopy. In comparison to mitochondrial and ER membranes, the peroxisomal membranes were slightly more disordered and characterized by a distinct enrichment of phosphaditylinositol, indicating an important role of this phospholipid in peroxisomal membrane associated processes.

## Introduction

Poikilothermic organisms such as bacteria, fungi, reptiles, and fish cannot control their body temperature and must adapt their membrane lipid composition in order to maintain the fluidity of membranes at different temperatures. Surprisingly, little is known about the regulation of membrane fluidity in different organelles and especially in response to cellular stress (Ballweg and Ernst, 2017). The yeast *Saccharomyces cerevisiae* has widely been used to study the architecture and regulation of lipid metabolism in eukaryotes. Growth of *S. cerevisiae* on oleic acid causes environmental stress that leads to a massive induction of expression of enzymes of fatty acid beta-oxidation. In fungi, the enzymes for beta-oxidation are exclusively localized in peroxisomes and growth on oleic acid results in a massive proliferation of these organelles (Veenhuis et al., 1987).

Peroxisomes are ubiquitous, dynamic organelles of eukaryotic cells. Depending on the organism, the tissue and environmental conditions, peroxisomes differ in morphology and vary in size between 0.1 to 1.0 μm in diameter (van den Bosch et al., 1992). The organelle is surrounded by a single lipid bilayer and enclosures a crystalline protein-rich matrix. Peroxisomes are involved in a multitude of metabolic processes beside the oxidation of fatty acids, including the depletion of hydrogen peroxide, which is the eponymous reaction of peroxisomes. Defects in peroxisome biogenesis or function leads to severe diseases such as the Zellweger syndrome (Nagotu et al., 2012; Waterham et al., 2015). In contrast to cell organelles like mitochondria or chloroplasts, peroxisomes do not contain their own genome. Hence, peroxisomal matrix proteins are nuclear encoded, and synthetized on free ribosomes. The newly synthesized proteins are recognized by specific import receptors in the cytosol, directed to the peroxisomal membrane and imported into the peroxisomal lumen via specific transient import pores (Meinecke et al., 2010; Montilla-Martinez et al., 2015). Pex5p is the import receptor for proteins harboring peroxisomal targeting signals of type 1 (PTS1) and the import pore for PTS1-proteins mainly consists of the Pex5p and its membrane bound docking protein Pex14p. Accordingly, proteins to be imported bind to Pex5p via their PTS1 and the cargo-loaded Pex5p is then directed to Pex14p at the peroxisomal membrane, where the originally cytosolic Pex5 gets integrated into the peroxisomal membrane and functions as an integral part of the translocation pore (Meinecke et al., 2016). The mechanism of binding and integration of Pex5p into the peroxisomal membrane is still a matter of debate. On one hand, Pex5p is known to directly bind to the peroxisomal membrane proteins Pex13p and Pex14p (Elgersma, 1996; Erdmann, 1996; Fransen et al., 1998; Huhse et al., 1998; Girzalsky et al., 1999; Barnett, 2000; Stein et al., 2002). On the other hand, Pex5p can also integrate spontaneously into phospholipid membranes, independent of Pex13p and Pex14p (Kerssen et al., 2006). Related to this, the functions in different peroxisomal metabolic pathways require its membrane to be permeable for respective solutes. Here, to date several transporters and water-filled channels have been identified in peroxisomal membranes from different organisms (Shani and Valle, 1996; Palmieri et al., 2001; Rokka et al., 2009; Mindthoff et al., 2016).

The function of all of the aforementioned integral or associated membrane proteins is not only dependent on the interplay with other proteins but also with lipids. Besides direct lipid binding sites as identified for different peroxisomal proteins (Shiozawa et al., 2006), the characteristics of the lipid membrane environment such as lipid membrane ordering or fluidity and lipid membrane composition is generally an important factor for membrane protein functionality. It determines efficiency of membrane binding and integration of proteins, as well as organization, mobility and interaction of proteins in and at the membrane (Sezgin et al., 2017a). Therefore, detailed knowledge of both lipid membrane ordering and lipid membrane composition is of utmost importance for the understanding of protein function and for interference with malfunctions.

Analysis of the lipid membrane content and lipid membrane ordering is straightforward via lipidomics and fluorescence microscopy. Lipidomics involves the determination of the complete lipid profile (the lipidome) within a cell or tissue sample using mass spectrometer (MS) approaches such as Thin Layer Chromatography (TLC) and electrospray ionization mass spectrometry (ESI MS) (Shevchenko and Simons, 2010; De Kroon, 2017), while the quantification of shifts in the fluorescence spectrum of membrane-incorporating dyes such as Laurdan are for example employed to determine membrane lipid order or fluidity (Parasassi et al., 1998; Sezgin et al., 2015). Previous studies of general cell lipid contents have for example identified peroxisomal-disease related changes in phospholipid ratios in fibroblast cells from Zellweger patients (Herzog et al., 2016), the requirement for peroxisomal function during influenza virus replication (Tanner et al., 2014), or the role of certain lipids in pexophagy (Grunau et al., 2011) or peroxisome-mediated cholesterol transport (Chu et al., 2015).

The measurements of lipid order or contents of peroxisomes require their accurate isolation from cells. Here, *S. cerevisiae* has developed as a useful model system (Dickson, 2008; Klug and Daum, 2014; Kohlwein, 2017a). Upon growth on oleic acid medium, the abundance of peroxisomes is massively increased, which allows the efficient isolation of peroxisomal membranes. A striking issue with measuring lipid membrane characteristics of peroxisomes is the dependence on culturing conditions. Fatty acids in yeast cells can be synthesized *de novo* or salvaged from the medium (Koch et al., 2004). It was shown that acyl chain composition of organelle membranes changes dramatically if yeast is cultured in fatty acid-containing medium in contrast to glucose-grown cells, which resulted in an incorporation of fatty acids provided in the medium in organelle membranes (Wriessnegger et al., 2007; Rosenberger et al., 2009). Similarly to variations of the growth conditions differences in the genotype of yeast strains can lead to variances in the lipid composition of subcellular compartments (Klose et al., 2012). Consequently, it is important to accurately control culturing and genotype conditions when comparing lipid characteristics between organelles.

Here we investigated the lipid composition and lipid order of peroxisomal membranes of oleic acid-induced cells directly with corresponding mitochondrial and ER membranes. Isolation of organelles was performed in parallel by differential and density gradient centrifugation and prepared membranes were subjected to shotgun lipidomics for lipid composition analysis and to fluorescence microscopy using a polarity sensitive membrane dye for lipid order characterization. In comparison to mitochondrial and ER membranes, peroxisomal membranes were more disordered and thus more fluid and characterized by a significant enrichment of PI. Our results will help interpreting existing and future data on peroxisomal membrane organization, signaling and biogenesis.

## Materials and Methods

### Yeast Strain and Culture Conditions

The *S. cerevisiae* strain used in the present study was UTL-7A wild-type (*MATa*, *leu2-3*, and *112ura3-52 trp1*) (Erdmann et al., 1989). YPD medium contained 2% (w/v) glucose, 2% (w/v) peptone, and 1% (w/v) yeast extract. YNBG medium contained 0.17% (w/v) yeast nitrogen base without amino acids, 0.1% (w/v) yeast extract, and 5% (w/v) ammonium sulfate, adjusted to pH 6.0. YNBGO medium contained 0.1% (w/v) instead of 0.3% (w/v) glucose and in addition 0.1% (v/v) oleic acid (Applichem, Darmstadt, Germany), and 0.05% (v/v) Tween 40. It has to be noted, that oleic acid used for media contained impurities. In detail we used oleic acid of a purity of 65 – 88% with maximum limit of imporities of myristic acid (5%); palmitic acid (16%); stearic acid (6%); linoleic acid (18%); linolenic acid (4%), and fatty acids with chain length greater than C18 (4%). All cultures were grown at 30°C. Cells from a glycerol-culture were plated onto a fresh YPD-plate [YPD medium containing 2% (w/v) select-agar] and grown for 3 days. Plated cells were used to inoculate 20 ml of YNBG grown for 8 h. After adding 30 ml of fresh medium cells were grown for 16 h. This culture was used to inoculate 1.25 L YNBGO medium to an OD_600*nm*_ of 0.1, which was further grown over night to mid logarithmic phase (OD_600*nm*_ of 2.6 – 2.8).

### Subcellular Fractionation and Isolation of Peroxisomes, Mitochondria, and Endoplasmic Reticulum

Yeast cells (UTL-7A) were grown in YNBGO medium and converted to spheroplasts as described previously (Cramer et al., 2015). In brief, harvested cells were washed once with sterile water centrifuging at 5,000 × *g* (Allegra^®^ X-15R, rotor: SX4750A) for 5 min. Further, cells were resuspended in five-fold volume DTT buffer (100 mM Tris, 10 mM DTT) of the wet-weight and incubated at 30°C at 60 rpm for 20 min. Then, cells were sedimented for 7 min (Allegra^®^ X-15R, rotor: SX4750A, 600 × *g*) and washed twice with 1.2 M sorbitol dissolved in sterile water (sorbitol-solution). Sedimented cells were resuspended in five-fold volume sorbitol buffer (1.2 M sorbitol, 20 mM K_3_PO_4_, adjusted to pH 7.4 with 1 M KH_2_PO_4_) of the wet weight and 1,000 units/g cells lyticase (Sigma-Aldrich) were added. Enzymatic digestion was performed at 37°C at 60 rpm for approximately 20 min and stopped when OD_600*nm*_ of the cells reached 10% of the starting value. All further steps were performed on ice or at 4°C. After washing twice with sorbitol-solution, the spheroplasts were left to swell for 20 min in breaking buffer (5 mM MES, 1 mM KCl, 1 mM EDTA, and 1 μg/ml Antipain, Aprotinin, Bestatin, Chymostatin, Leupeptin, Pepstatin, 10 μg/ml PMSF; adjusted to pH 6.0 with KOH). A cell free homogenate and the corresponding post-nuclear supernatant (PNS) were obtained by gentle breakage of spheroplasts with a dounce homogenizer and subsequent sedimentation according to (Cramer et al., 2015). Separation of cellular organelles was obtained by isopycnic density gradient centrifugation of 20 mg of the PNS, in a linear 2.24% to 24% (w/v) OptiPrep^TM^ (Iodixanol; Axis-Shield PoC AS, Oslo, Norway)/18% (w/v) sucrose gradient. Resulting fractions were analyzed by immunoblotting using markers for different organelles. Peroxisomal fractions were identified enzymatically by monitoring the activity of peroxisomal catalase and by immunoblot analysis. Highly enriched peroxisomes were obtained after combining peroxisomal peak fractions, dropwise dilution with five-fold volume of breakage buffer (without protease inhibitors) and concentration of the organelles by sedimentation (Beckman Coulter rotor 70 Ti, 100,000 × *g*, 30 min, 4°C) onto a 2 M sucrose cushion.

Mitochondrial and ER-containing fractions of gradients were combined and sedimented in the same way as described for peroxisomes. The concentrated organelles (mostly mitochondria and endoplasmic reticulum) were further subjected to a three-step sucrose gradient centrifugation (1.5 ml 60%, 4 ml 32%, 1.5 ml 23%, and 1.5 ml 15%) as described in (Meisinger et al., 2000). Resulting fractions were analyzed by immunoblotting for specific organelle markers. Mitochondrial and endoplasmic reticulum membranes were concentrated by sedimentation as described for peroxisomes, except that SEM-buffer (250 mM sucrose, 1 mM EDTA, 10 mM MOPS, pH 7.2) was used. For lipidomic analysis, concentrated peroxisomes, mitochondria and endoplasmic reticulum were treated twice with 150 mM NH_4_HCO_3_ (Beckman Coulter rotor MLA-130, 186,000 × *g*, 45 min, 4°C). Sedimented membranes were diluted in 150 mM NH_4_HCO_3_, protein concentration was determined with an UV–VIS spectrophotometer (NanoDrop 1000, Peqlab, Erlangen, Germany), and membranes were adjusted to a final concentration of 0.25 mg/ml in 100 μl volume. For GP-measurements, concentrated membranes were used directly after enrichment without further treatment. Samples were snap-frozen in liquid nitrogen and stored at −80°C until subsequent analysis. The enrichment and purity of isolated membranes was controlled by immunoblot analysis and silver-staining of the polyacrylamide gels as described (Blum et al., 1987).

### Preparation of Yeast Cell Lysates

From the same yeast main culture used for isolation of organelles, a portion corresponding to 20 OD_600*nm*_ units/1 ml was treated as described previously (Klose et al., 2012). In particular, cells were sedimented by centrifugation at 5,000 × *g* for 3 min (Allegra X-15R, rotor SX4750A, 20°C) and washed twice with 150 mM NH_4_HCO_3_. Cell lysis of 1 ml of the suspension was performed adding an equal volume of the wet weight of glass beads (0.5–0.7 mm diameter) and vortexing for 10 min with alternating cooling periods on ice for 1 min. 100 μl of the total yeast lysate (L) was snap-frozen in liquid nitrogen and stored at −80°C for determination of the total yeast lipid composition.

### Immunodetection

The isolation and purity of the organelle membranes was monitored by immunoblot analysis with polyclonal rabbit antibodies raised against Pcs60p (Blobel and Erdmann, 1996), Cta1p and Fox1p (Schafer et al., 2004), Tom40p and Tim23p (from K. Pfanner, University of Freiburg), Por1p and Kar2p (from R. Rachubinski, University of Alberta), Fbp1p (Entian et al., 1988) and mouse antibodies raised against Dpm1p (Invitrogen, Karlsruhe, Germany). Primary antibodies were detected with an IRDye 800CW goat anti-rabbit or anti-mouse IgG secondary antibody (Li-Cor Bioscience, Bad Homburg, Germany).

### Membrane Fluidity-Measurements

Membrane fluidity-measurements were performed as described (Sezgin et al., 2015). Enriched organelle membranes, present in breaking buffer, were labeled with the fluorescent dye Di-4-ANEPPDHQ (Thermo Fisher Scientific, Waltham, MA, United States) (Jin et al., 2006) at a final concentration of 0.4 μM at room temperature. Subsequently, the prepared membranes were monitored by spectral imaging on a Zeiss LSM 780 confocal microscope equipped with a 32-channel GaAsP detector array. The lambda detection range was set between 415 and 691 nm as ultimate limits for FE. Despite the fact that wavelength intervals of down to 4 nm could be chosen for the individual detection channels, we have set these intervals to 8.9 nm, which allowed the simultaneous coverage of the whole spectrum with the 32 detection channels. The images were saved in. LSM file format and then analyzed to calculate General Polarization (GP) values by using a custom plug-in compatible with Fiji/ImageJ, as described previously (Sezgin et al., 2015).

### Lipid Extraction for Mspectrometry Lipidomics

Mass spectrometry-based lipid analysis was performed at Lipotype GmbH (Dresden, Germany) as described (Ejsing et al., 2009; Klose et al., 2012). Lipids were extracted using a two-step chloroform/methanol procedure (Ejsing et al., 2009). Samples were spiked with internal lipid standard mixture containing: CDP-DAG 17:0/18:1, ceramide 18:1;2/17:0 (Cer), stigmastatrienol, diacylglycerol 17:0/17:0 (DAG), lyso-phosphatidate 17:0 (LPA), lyso-phosphatidylcholine 12:0 (LPC), lyso-phosphatidylethanolamine 17:1 (LPE), lyso-phosphatidylinositol 17:1 (LPI), lyso-phosphatidylserine 17:1 (LPS), phosphatidate 17:0/14:1 (PA), phosphatidylcholine 17:0/14:1 (PC), phosphatidylethanolamine 17:0/14:1 (PE), phosphatidylglycerol 17:0/14:1 (PG), phosphatidylinositol 17:0/14:1 (PI), phosphatidylserine 17:0/14:1 (PS), ergosterol ester 13:0 (EE), triacylglycerol 17:0/17:0/17:0 (TAG), inositolphosphorylceramide 44:0;2 (IPC), mannosyl- inositolphosphorylceramide 44:0;2 (MIPC), and mannosyl-di-(inositolphosphoryl)ceramide 44:0;2 (M(IP)_2_C). After extraction, the organic phase was transferred to an infusion plate and dried in a speed vacuum concentrator. 1st step dry extract was re-suspended in 7.5 mM ammonium acetate in chloroform/methanol/propanol (1:2:4, V:V:V) and 2nd step dry extract in 33% ethanol solution of methylamine in chloroform/methanol (0.003:5:1; V:V:V). All liquid handling steps were performed using Hamilton Robotics STARlet robotic platform with the Anti Droplet Control feature for organic solvents pipetting.

### MS Data Acquisition

Samples were analyzed by direct infusion on a QExactive MS (Thermo Scientific) equipped with a TriVersa NanoMate ion source (Advion Biosciences). Samples were analyzed in both positive and negative ion modes with a resolution of *R*_*m/z* = 200_ = 280,000 for MS and *R*_*m/z* = 200_ = 17500 for MSMS experiments, in a single acquisition. MSMS was triggered by an inclusion list encompassing corresponding MS mass ranges scanned in 1 Da increments (Surma et al., 2015). Both MS and MSMS data were combined to monitor EE, DAG and TAG ions as ammonium adducts; PC as an acetate adduct; and CL, PA, PE, PG, PI, and PS as deprotonated anions. MS only was used to monitor LPA, LPE, LPI, LPS, IPC, MIPC, and M(IP)_2_C as deprotonated anions; Cer and LPC as acetate adducts and ergosterol as protonated ion of an acetylated derivative.

### Data Analysis and Post-Processing

Data were analyzed with in-house developed lipid identification software based on LipidXplorer (Herzog et al., 2011, 2012). Data post-processing and normalization were performed using an in-house developed data management system. Only lipid identifications with a signal-to-noise ratio >5, and a signal intensity five-fold higher than in corresponding blank samples were considered for further data analysis. Lipid identifiers of the SwissLipids database (Aimo et al., 2015)^1^ are provided in Supplementary Table 1.

### Nomenclature

The following lipid names and abbreviations were used; SP, sphingolipids; include: Cer, ceramides; IPC, inositolphosphorylceramide; MIPC, mannosyl-inositol phosphorylceramide; M(IP)2C, mannosyl-di-(inositolphosphoryl) ceramide; GP, glycerophospholipids; include: PA, phosphatidic acid; PC, phosphatidylcholine; PE, phosphatidylethanolamine; PI, phosphatidylinositol; PS, phosphatidylserine; CL, cardiolipin; and their respective lysospecies: lysoPA, lysoPC, lysoPE, lysoPI, and lysoPS; SL, sphingolipids; GL, glycerolipids; include: DAG, diacylglycerol; TAG, triacylglycerol; Sterols include: Erg, ergosterol; Lipid species are annotated according to their molecular composition. Glycero- and glycerophospholipid species are annotated as: <lipid class><sum of carbon atoms in the fatty acids>:<sum of double bonds in the fatty acids> (e.g., PI 34:1). Sphingolipid species are annotated as: <lipid class><sum of carbon atoms in the long chain base and fatty acid moiety>:<sum of double bonds in the long chain base and the fatty acid moiety>:<sum of hydroxyl groups in the long chain base and the fatty acid moiety> (e.g., IPC 44:0;4).

## Results

### Isolation of Peroxisomal, Mitochondrial, and ER Membranes

**FIGURE 1:**
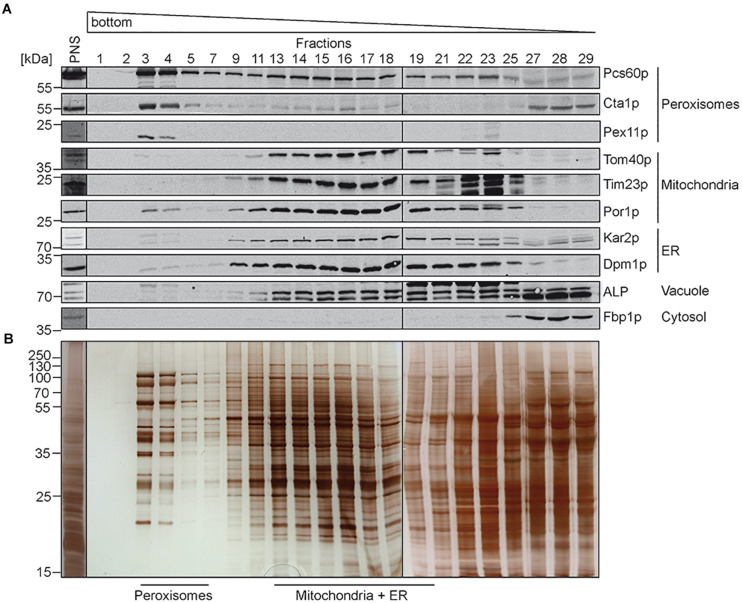
Isolation of peroxisomes by isopycnic density gradient centrifugation. Cells of *S. cerevisiae* wild-type strain were grown under oleate-inducing conditions for 16 h. A post nuclear supernatant (PNS) was prepared, and organelles were separated on an OptiPrep^TM^/sucrose gradient. Fractions were collected from the bottom of the gradient. Equal volumes of the indicated gradient fractions were separated by SDS-PAGE and analyzed by **(A)** immunoblotting using antibodies against Pcs60p (peroxisomal matrix protein), Cta1p (catalase; peroxisomal matrix protein), Pex11p (peroxisomal membrane protein), Tom40p and Por1p (mitochondrial outer membrane protein), Tim23p (mitochondrial inner membrane protein), Kar2p (ER luminal protein), Dpm1p (ER membrane protein), ALP (vacuole), and Fbp1p (cytosol), or by **(B)** silver staining. Peroxisome-containing fractions (3–5) were pooled, enriched and used for fluidity and lipidomic analysis. Fractions of mitochondria and ER containing membranes (13–19) were processed for further purification (Figure 2).

In previous studies, comparative determination of lipid characteristics in organelle membranes were performed from organelles isolated from yeast cells grown under different conditions. As outlined above, it is well known that beside the genotype, growth phase, and temperature, also the carbon source in the growth-medium (e.g., glucose, ethanol, and oleic-acid) can dramatically change the lipid composition of cellular membranes (Klose et al., 2012). Here we compared the lipid composition of isolated different organelles from the same *S. cerevisiae* strain grown in medium containing oleate as sole carbon source. To this end, highly purified peroxisomal, mitochondrial and ER membranes were obtained from oleate-induced wild-type *S. cerevisiae* cells by a combined approach of differential and isopycnic Optiprep density gradient centrifugation. The density gradient for the separation of peroxisomes from other organelles is shown in Figure 1. Fractions were collected from the bottom to the top of the gradient and analyzed by immunoblotting (Figure 1A). In such gradients, the peroxisomal matrix protein Pcs60p localizes to cytosolic fractions, due to organelle breakage and to peroxisomes (Blobel and Erdmann, 1996), which peak in fractions 3 and 4. These peaks also contained the fractions of the peroxisomal membrane protein Pex11p and the peroxisomal matrix protein catalase (Cta1p). The data show that the majority of peroxisomes localized to fractions 3 and 4, which exhibit a high density of 1.21 g/cm^3^ as typical for mature peroxisomes (Graham et al., 1994). The peroxisomal peak fractions only showed minor contaminations with mitochondrial markers (Tom40p, Tim23p, and Por1p), the ER-markers Kar2p and Dpm1p, or alkaline phosphatase (ALP), the vacuolar marker. These markers were most prominent in lighter fractions, well separated from the peroxisomal fractions. To gain more insight into the degree of enrichment of peroxisomal membranes, we performed signal intensity measurements of immunoblots depicted in Figure 1 (Supplementary Figure 2). While Pcs60p was found with 23% in fractions 3+4, it spreads over the entire gradient fractions, most likely by association with other membrane after organelle disruption, the soluble peroxisomal catalase (Cta1p) displays two peaks, one in the peroxisomal (20% in fractions 3+4) and another in soluble fraction. We consider the integral membrane protein Pex11p as suitable marker for localization of peroxisomal membranes. It turned out that 83% of total Pex11p (with the sum signals of all fractions set to 100%) is located in fractions 3+4 of the gradient, in line with successful enrichment of these membranes. These fractions just contain 0.78% mitochondrial Tom40p (4.3% Por1p) and 1.26% of the ER-protein Dpm1p (Supplementary Figure 2). The data indicate that peroxisomes were purified with great homogeneity, which is also supported by the distinct protein pattern of fractions 3 to 4 (Figure 1B) that is typical for purified oleic acid-induced peroxisomes (Erdmann and Blobel, 1995).

**FIGURE 2:**
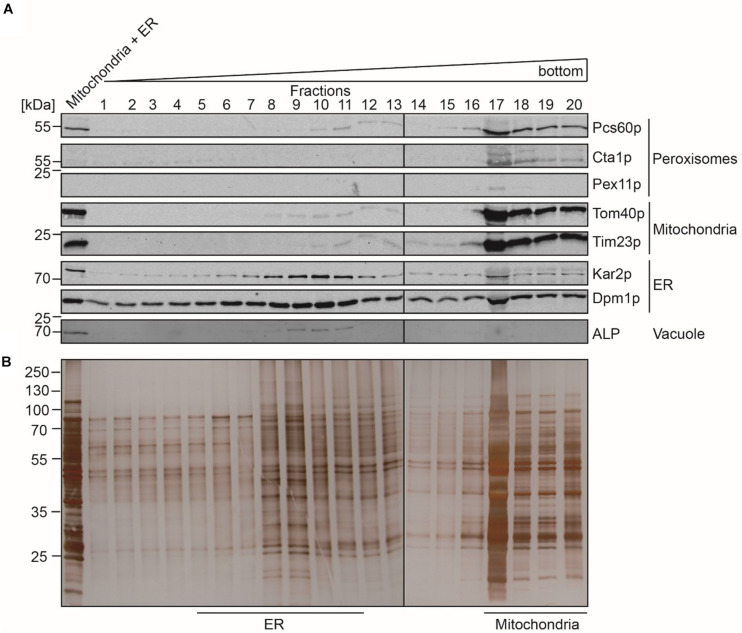
Separation of mitochondria and ER by sucrose density gradient centrifugation. Enriched fractions 13–19 (mitochondria + ER) gained after the first isopycnic density gradient centrifugation were loaded onto a dicontinuous sucrose gradient. Fractions were collected from the top of the gradient. Equal volumes of the indicated gradient fractions (fraction 17 diluted 1:1) were separated by SDS-PAGE and analyzed by **(A)** immunoblotting using antibodies against Pcs60p (peroxisomal matrix protein), Cta1p (catalase; peroxisomal matrix protein), Pex11p (peroxisomal membrane protein), Tom40p and Por1p (mitochondrial outer membrane protein), Tim23p (mitochondrial inner membrane protein), Kar2p (ER luminal protein), Dpm1p (ER membrane protein), and ALP (vacuole), or by **(B)** silver staining. ER-peak fractions (5–11) and peak fractions of mitochondria (17–20) were used for lipidomic and fluidity measurements.

In contrast to peroxisomes, the Optiprep density gradient centrifugation did not lead to a sufficient purification of mitochondria and the ER. We therefore performed a second purification step, for which the fractions that contained the highest content of mitochondrial and ER protein-markers (Figure 1A, fractions 13 to 19) were pooled and organelles concentrated by sedimentation. The resulting sample (Figure 2A, Load) contained mainly mitochondria and ER but also minor amounts of peroxisomes and vacuoles as indicated by the presence of the peroxisomal Pcs60p and the vacuolar ALP. However, the peroxisomal membrane marker Pex11p and peroxisomal catalase were below the detection limit. The pooled sample was separated by a three-step sucrose density gradient centrifugation. Fractions were collected from top of the gradient, and equal amounts were analyzed by immunoblotting (Figure 2A). Most of the mitochondria were found in fractions 17 to 20 with the highest protein content in fraction 17 (Figure 2B). The detection of the mitochondrial outer membrane protein Tom40p and the mitochondrial inner membrane protein Tim23p in the same fractions indicated that the isolated mitochondria were still intact. The mitochondrial peak fractions also contained minor portions of peroxisomal Pcs60p, ER-marker proteins Kar2p (luminal protein) and Dpm1p. However, the mitochondrial peak fraction was well separated from the ER-peak at fractions 5 to 11 (Figure 2A). The ER-peak co-segregated with the loaded vacuolar marker but showed nearly no contamination with peroxisomes or mitochondria.

**FIGURE 3:**
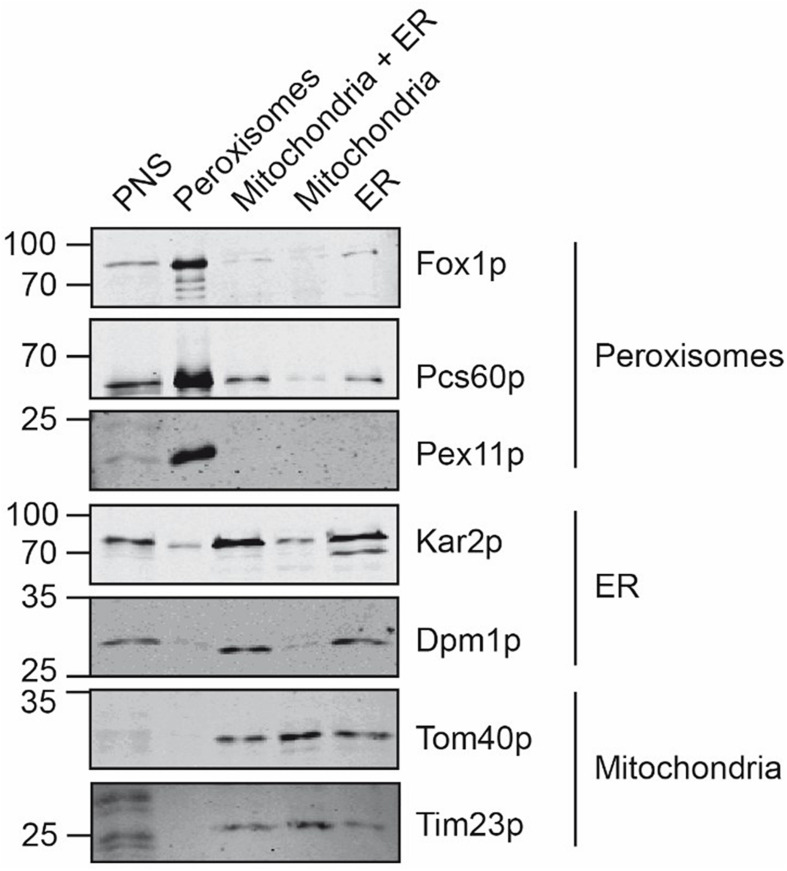
Comparative analysis of isolated peroxisomes, mitochondria and ER. Equal amounts (20 μg) of the postnuclear supernatant, purified peroxisomes, the mitochondria/ER pool after the first purification step, as well as purified mitochondria and ER were separated by SDS-PAGE and analyzed by immunoblotting using antibodies against Fox1p (peroxisomal matrix protein), Pcs60p (peroxisomal peripheral membrane and matrix protein), Pex11p (peroxisomal membrane protein), Kar2p (ER luminal protein), Dpm1p (ER membrane protein), Tom40p (mitochondrial outer membrane protein) and Tim23p (mitochondrial inner membrane protein).

The organelles of the peroxisomal, mitochondrial and ER peak fractions were pooled, concentrated by sedimentation, and further processed in parallel. To estimate the purity of the isolated organelles, equal amounts of the starting material (PNS) and the isolated organelles were analyzed by immunoblotting using organelle-specific marker-proteins (Figure 3). The enriched peroxisomes contained the peroxisomal Pcs60p, Pex11p, and Fox1p, appearing as a significant signal in the immunoblot but they were devoid of contaminating mitochondria- and ER-membrane marker. Likewise, the isolated mitochondria did not contain peroxisomal membrane marker Pex11p or the ER-membrane marker Dpm1p, suggesting that the isolated mitochondrial membranes were of sufficient purity. Despite the observation that ER fractions did not contain appreciable amounts of contaminating organelles after sucrose density gradient fractionation, the sedimented ER-fraction contained significant amount of the mitochondrial membrane-marker-proteins Tom40p and Tim23p, but were free of peroxisomal membrane indicated by lack of Pex11p-detection.

### Fluidity

**FIGURE 4:**
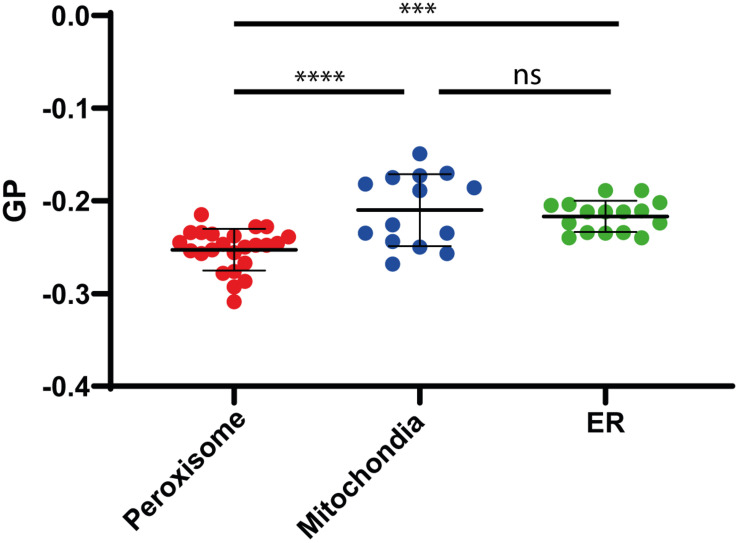
Membrane fluidity. Generalized polarization (GP) values measured on isolated Peroxisomes (red), Mitochondria (blue), and ER (green), indicating a slightly higher fluidity (lower GP value) for peroxisomal membranes, compared to mitochondria and ER membranes. Shown are individual measurements and the mean values with standard deviation from the three independent experiments. Statistical analysis: Ordinary One-way ANOWA with multiple comparisons; ****P < 0.0001; ***P = 0.0003; ns: not significant.

To analyze the lipid membrane order, we employed the polarity-sensitive, membrane-embedded fluorescent dye Di-ANEPPDHQ, which experiences a shift in fluorescence emission spectrum depending on the molecular order of the membrane environment. This shift can be quantified by a General Polarization (GP) parameter, which we calculated from the whole emission spectrum recorded on our confocal microscope (spectral imaging, see materials and methods), and whose values are negative for rather fluid disordered and increase for more ordered membrane environments (Sezgin et al., 2015). Performing spectral GP analysis on the isolated organelles resulted in statistically accurate average values GP < −0.2 (Figure 4), which indicate a rather fluid and disordered lipid membrane environment when compared to the intact plasma membrane of living cells (GP > 0–0.3) (Sezgin et al., 2015, 2017b). However, compared to mitochondrial or ER membrane (GP = −0.22), the peroxisomal membrane was slightly more fluid (GP = −0.25) (Figure 4). The difference in the GP value is significant as shown by a one-way ANOVA (Peroxisome v. mitochondria *P* < 0.0001; Peroxisome v ER P 0.0003; mitochondria v. ER not significant).

### Shotgun Lipidomics

**FIGURE 5:**
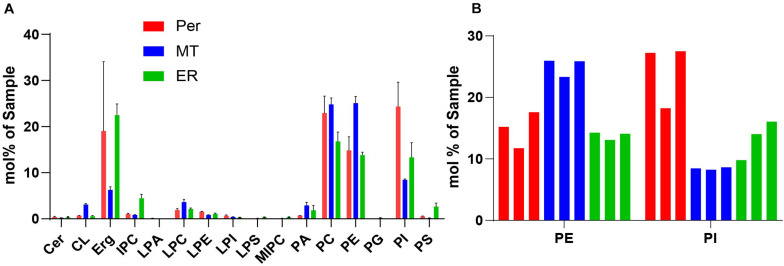
**(A)** Selected lipidomics data of isolated organelles. Peroxisomes (red, Per), mitochondria (blue, MT), and ER (green), indicating generally high levels of phospholipids (PC, PE, and PI), and in comparison, increased levels of PI for peroxisomes. Shown are mean values with standard deviation from three indipendent experiments. For more detailed lipidomics data see Supplementary Figure 1. **(B)** Lipidomic data of PE and PI in Peroxisomes, Mitochondra and ER were from three independent experiments. Mole percentages of the total amount of lipids are shown on the *y*-axis (sum amount of a lipid class divided by the total amount in the sample, multiplied by 100).

**FIGURE 6:**
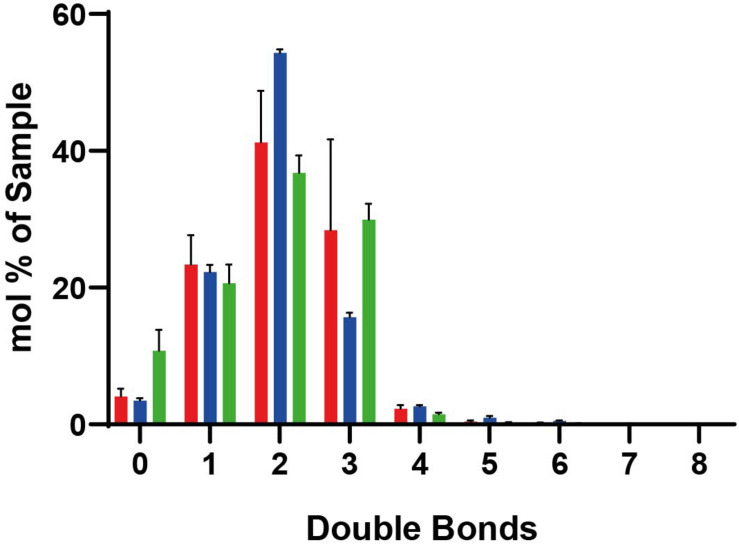
Lipidomic data for lipids with different numbers of double bounds. In peroxisomes (red), mitochondria (blue), and ER (green). Standard deviations result from the three different measurements. Mole percentages of the total amount of lipids are shown on the *y*-axis (sum amount of a lipid species with the same double bond count divided by the total amount in the sample, multiplied by 100).

We performed shotgun lipidomics (Klose et al., 2012) on the isolated peroxisomes (Per), mitochondria (MT) and ER for comparison of the lipid composition of these organelles and correlation with the estimated lipid order characteristics. Figure 5 shows the relative amount of some prominent lipids, Figure 6 shows the distribution of unsaturated lipids between the organelles, while Supplementary Figure 1 gives a more detailed overview over all lipids investigated. Clearly, all organelle membranes were dominated by ergosterol (Erg) and the phospholipids phosphatidylcholine (PC), phosphatidylethanolamine (PE), and phosphatidylinositol (PI), in principle confirming previous lipidomics studies (Zinser et al., 1991; Schneiter et al., 1999; Tuller et al., 1999; Rosenberger et al., 2009). The following most pronounced differences between the different organelle membranes arose: MT – a relative enrichment of the typical mitochondrial lipid cardiolipin (CL) and of the phospholipids PE, PC and its lyso-form lyso-PC (LPC), as well as a relative decreased level of Erg; ER – a relatively increased amount of the sphingolipids (SP) inositolphosphorylceramide (IPC), mannosyl-inositol phosphorylceramide (MIPC), and the phospholipid PS, as well as relatively reduced levels of PC; Per – an increased level of PC (similar to that of MT) and a distinct relative high level of PI, as well as a relative low level of phosphatidic acid (PA).

## Discussion

Here, we present for the first time a detailed quantitative, mass spectrometry-based comparative analysis of the membrane lipidome and lipid order of yeast peroxisomes, mitochondria and the ER under oleic acid-inducing growth conditions. While lipid order has so far not been determined for oleic acid-induced peroxisomal membranes, the lipid content of peroxisomes in rat liver and *S. cerevisiae* has been investigated previously (Zinser et al., 1991; Jeynov et al., 2006; Schrader et al., 2020). TLC and MS revealed that peroxisomal membranes are mainly composed of the PC, PE, PA, PS, and PI (Zinser et al., 1991; Schneiter et al., 1999; Tuller et al., 1999; Rosenberger et al., 2009). Furthermore, the acyl chain composition of lipids in *S. cerevisiae* is rather simple due to the lack of synthesis of polyunsaturated fatty acids. Mainly mono unsaturated acyl chain palmitoleic acid (C16:1) and oleic acid (C18:1) as well as the saturated palmitic acid (C16:0) and stearic acid (C18:0) are present (Schneiter et al., 1999). Comparison to the lipidome of other organelles such as mitochondria have revealed no significant differences for *S. cerevisiae* (Zinser et al., 1991; Tuller et al., 1999), in contrast to enhanced levels of PI in peroxisomes of yeast *Pichia pastoris* (Wriessnegger et al., 2007). A significant difference in the experimental design, which might attribute to a different outcome of our study is the fact that for these studies peroxisomal membranes from oleic acid grown cells were compared to ER and mitochondrial membranes from cells grown in galactose medium (Zinser et al., 1991). It is known that slight environmental changes may already result in variations of the total lipid compositions of the yeast (Kohlwein, 2017b). This is considered in our study, which is based on the parallel isolation and analysis of peroxisomes, mitochondria and the ER from the same cells. The study is emphasized for oleic-acid growth conditions, which results in a massive proliferation of peroxisomes (Veenhuis et al., 1987). However, growth in oleate containing media does not only boost the enlargement and multiplication of peroxisomes but also influences the lipid composition of all cellular lipids, since oleate is a building block for complex membrane lipids, especially phospholipids (Wriessnegger et al., 2007). However, the isolation of organelle membranes from the same cells can lead to cross-contamination. We obtained peroxisomal membranes with only tiny cross-contamination of ER- and mitochondrial membranes. Moreover, the isolated mitochondria contain only tiny portion of the ER-membrane marker Dpm1p and lack the peroxisomal membrane-marker Pex11p. Thus, we consider a high purity of these organelles (Figure 3). While the ER-membranes were devoid of peroxisomal membrane marker, significant portion of mitochondrial marker were found within this fraction (Figure 3). Probably this finding is due to membrane contact sites between ER and mitochondria also known as ER mitochondria encounter structure (ERMES) (for a review see Michel and Kornmann, 2012).

As we have a high enrichment of specific organelle markers in the analyzed fractions and find an enrichment of organelle specific lipids, like CL in mitochondria, we assume that the purity of our samples is sufficient to detect changes in their lipid composition and fluidity.

The applied shotgun lipidomics on isolated membranes from peroxisomes, mitochondria and ER of *S. cerevisiae* were reproducible between different batches (Figure 5). The data revealed characteristics of the different organellar membranes, such as a general high levels of ergosterol and phospholipids PC, PE, and PI in all organelles and the enrichment of the cardiolipin (CL) in mitochondria, as seen in previous studies (Zinser et al., 1991; Schneiter et al., 1999; Tuller et al., 1999; Rosenberger et al., 2009), these findings also underline a sufficient purity of our samples. However, features that were distinct between the organelles such as higher levels of PE and PC in mitochondria, of sphingolipid (M)IPC and PS in the ER, and PI in peroxisomes were also recognized. Moreover, reduced levels of ergosterol were detected in mitochondria, as well as less PC in the ER, and less PA in peroxisomes.

The comparison of lipidomes between different organelles is also of interest in light of peroxisomal biogenesis. In *S. cerevisiae*, peroxisomes can either arise by growth and division from pre-existing organelles (Lazarow and Fujiki, 1985) or by *de novo*-synthesis involving the endoplasmic reticulum (ER) (Hoepfner et al., 2005; Tam et al., 2005). Yet it is still a matter of debate, whether these processes happen in parallel or independent from each other, and in both cases, the main question of the origin of the peroxisomal membrane lipids remains partially unresolved. The ER is the organelle in eukaryotic cells, which synthesizes the majority of structural phospholipids such as PS or PI, cholesterol (ergosterol in yeast), non-structural triacylglycerol, and ceramides (the precursor for sphingolipids) (Van Meer et al., 2008). Peroxisomes do not contain enzymes for phospholipid biosynthesis and therefore rely on lipid trafficking mainly from the ER either by vesicular or non-vesicular transport (Levine, 2004; Holthuis and Levine, 2005; Raychaudhuri and Prinz, 2008; Lev, 2010; Lahiri et al., 2015). Therefore, a high similarity of the lipid composition of peroxisomes and the ER is to be expected and is seen in our study. Slight differences might arise from individual pathways. For example, PE can be synthesized at the mitochondrial membrane via PS that is derived from the ER (Tamura et al., 2012). This might explain the relatively high levels of PE and PS at the mitochondrial and ER membranes, respectively. Our analysis revealed PC as one of the most abundant lipids in oleic acid-induced mitochondria, which has been reported earlier for mitochondria derived from non-induced cells (Zinser et al., 1991; Schuler et al., 2016).

A striking feature that distinguishes the peroxisomal membrane from mitochondrial and ER membranes is the enhanced level of PI. The increased levels of PI may indicate that this lipid has specific functions for peroxisomes. Phosphorylated derivatives of PI are key regulators of many aspects of cellular physiology. In previous work, we have shown that Vps34p, which is the sole phosphatidylinositol-3-generating kinase in *S. cerevisiae*, localizes to peroxisomes and that the produced phosphatidylinositol-3 phosphate is required for pexophagy (Grunau et al., 2011). The functional importance of produced phosphatidylinositol-3 phosphate was shown in general, as the deletion of Vps34p blocked the autophagic breakdown of the peroxisomal marker enzyme Fox3p. Moreover, the role of peroxisomal produced phosphatidylinositol-3 phosphate was also demonstrated directly via the expression of the produced phosphatidylinositol-3 phosphate-binding motif FYVE-domain in WT cells. The EGFP-2xFYVE partially co-localized with the peroxisomal marker PTS2-DsRed in fluorescence microscopy experiments. This was not the case, when the binding-deficient version, EGFP-2xFYVE (C215S), was used. Importantly, the expression of EGFP-2xFYVE stabilized Fox3p in pexophagy assays, most likely by competing with produced phosphatidylinositol-3 phosphate -binding signaling proteins required for pexophagy, resulting in a block of pexophagy. The expression of the EGFP-2xFYVE (C215S) version did not interfere with pexophagy.

Our data indicate a rather fluid and disordered lipid membrane environment of peroxisomes, mitochondria and the ER. In comparison to mitochondria or the ER, peroxisomal membranes were slightly more fluid. In this respect, our data differ from an earlier study using a different approach based on anisotropy measurements (Zinser et al., 1991). The authors monitored the fluorescence of trimethylammonium diphenylhexatriene (TMA-DPH) that is fluorescent in membranes but not in water. The data indicated that peroxisomal membrane represents a rather rigid environment compared to mitochondria and microsomes. However, peroxisomes from oleate-induced cells were compared with organelles from cells grown in glucose medium, which might have an impact on membrane fluidity.

Key factors determining membrane fluidity are the proportion of saturated and unsaturated acyl chains in membrane lipids and the content of ergosterol. The Ole pathway that regulates expression of the single fatty acid desaturase Ole1p has been identified as a major mechanism to regulate membrane fluidity by determining the proportion of saturated and unsaturated acyl chains in membrane lipids (Ballweg and Ernst, 2017). Our lipidomic analysis of the peroxisomal membranes did not reveal a relative enrichment of lipids with a high number of double bounds or the presence of less ergosterol. Thus, there is no indication of an accumulation of unsaturated lipids or decrease in ergosterol as cause of the slightly increased disorder of peroxisomal membranes compared to mitochondria and ER. Therefore, other characteristics such as the distinct protein environment might be responsible for increased fluidity of the peroxisomal membrane.

Given the critical role of lipid compositions in determining organelle identity and function, it is clear that cells must adjust lipid metabolism during adaptive responses like the growth on oleic acid as single carbon source. The general low lipid membrane ordering in all three organelles may reflect the organelles membrane dynamics that warrant proper metabolite exchange, protein sorting and membrane trafficking. Of the observed differences in the lipid composition of the membranes of peroxisomes in comparison to mitochondria and ER from oleic acid-induced cells, the increased levels of PI in peroxisomes is most striking and may reflect the importance of this phospholipid for peroxisome function as precursor for signaling molecules regulating pexophagy.

## Data Availability Statement

The original contributions presented in the study are included in the article/Supplementary Material, further inquiries can be directed to the corresponding author/s.

## Author Contributions

LS performed the organelle isolation, ES and CK performed the lipidomic analyses. CE, RE, and KR designed the study or managed the project. KR, LS, ES, CK, HP, WG, CE, and RE analyzed the individual experiments. KR wrote the manuscript together with CE and RE with input of other authors. All authors discussed the results and commented on the manuscript.

## Conflict of Interest

CK was employed by the Lipotype GmbH. The remaining authors declare that the research was conducted in the absence of any commercial or financial relationships that could be construed as a potential conflict of interest.

## Footnotes

1 http://www.swisslipids.org

## Abbreviations

ALP: alkaline phosphatase
Cer: ceramides
CL: cardiolipin
DAG: diacylglycerol
Erg: ergosterol
GL: glycerolipids
GP: Generalized Polarization
GP: glycerophospholipids
IPC: inositolphosphorylceramide
M(IP)2C: mannosyl-di-(inositolphosphoryl) ceramide
MIPC: mannosyl-inositol phosphorylceramide
MS: mass spectrometry
PTS: peroxisomal targeting signal
TAG: triacylglycerol
TLC: thin layer chromatography
PA: phosphatidic acid
PC: phosphatidylcholine
PE: phosphatidylethanolamine
PI: phosphatidylinositol
PS: phosphatidylserine
SL: sphingolipids
SP: sphingolipids.
