# Supplementary material for: Fluidity and Lipid Composition of Membranes of Peroxisomes, Mitochondria and the ER From Oleic Acid-Induced Saccharomyces cerevisiae
Source: Front Cell Dev Biol. 2020 Oct 29;8:574363. doi: 10.3389/fcell.2020.574363 (PMC7658010; doi:10.3389/fcell.2020.574363)
Supplement: Supplementary file 1 [file Data_Sheet_1.docx]

**Supplementary Information**

**Fluidity and lipid composition of membranes of peroxisomes, mitochondria and the ER from oleic acid-induced Saccharomyces cerevisiae.**

Reglinski K. ^1,2,3,4^ *,Steinfort L.L.^5^*, Sezgin E.^3,6^, Klose C.^7^, Platta, H.W.^8^, Girzalsky, W.^5^, Eggeling C.^1,2,3,9^, Erdmann R.^5^


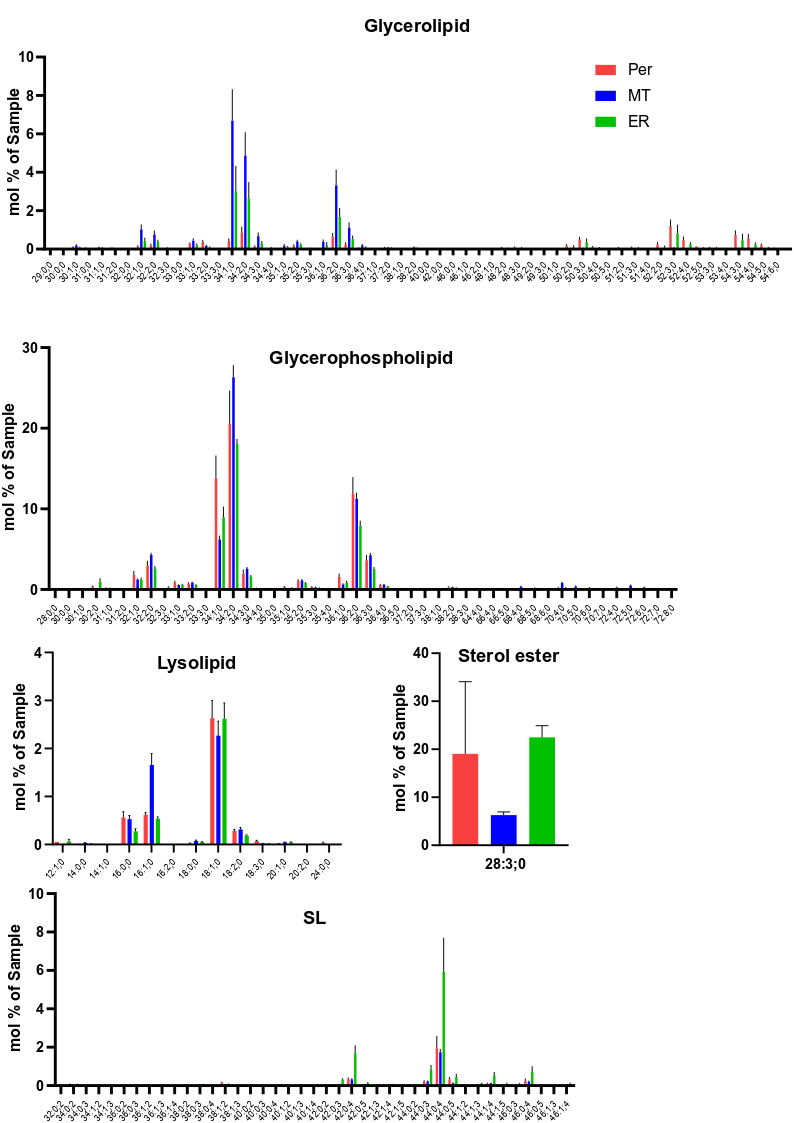


**Supplementary Figure 1: Complete lipidomic analysis of isolated organelles.** Peroxisomes (red), mitochondria (blue) and Er (green). Plotted are different lipid species (Glycerollipids, Glycerophospholipids, Lysolipids, Sterol ester and sphingolipids (SL) sorted by their category and sandardized on the amount of the lipids found in the individual samples. Shown are mean values with standard deviation from three independent experiments.


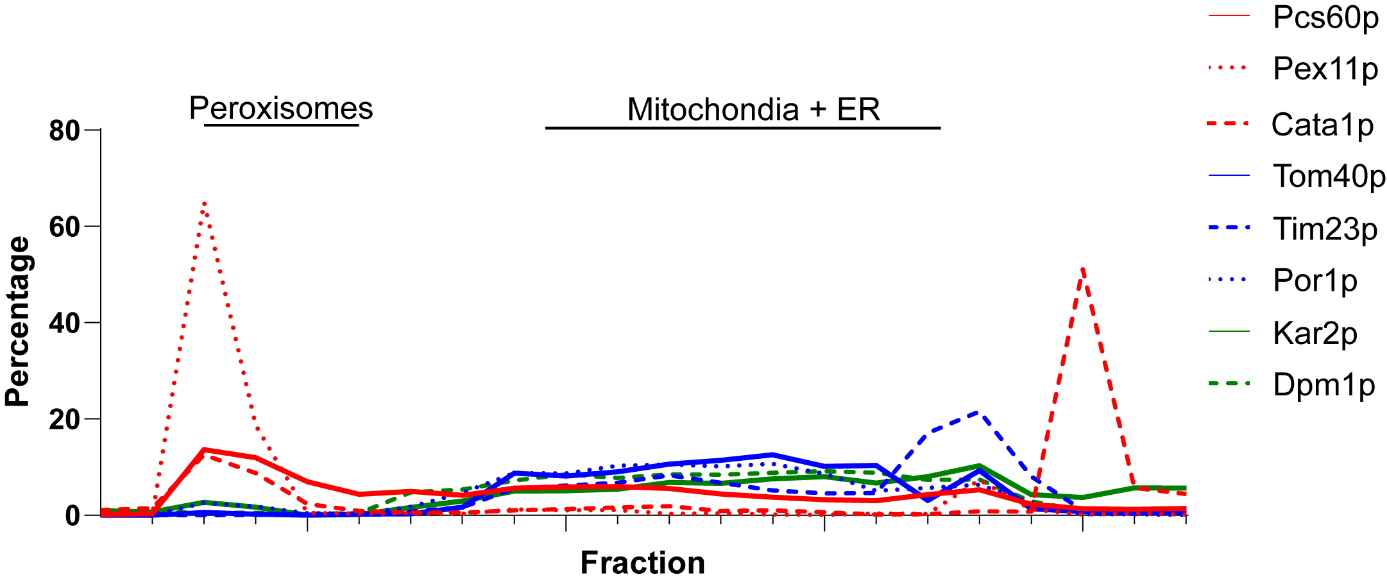


**Supplementary Figure 2: Densitometric analysis of the OptiPrep™ / sucrose gradient shown in Figure 1.** The gradient fractions were separated by SDS-PAGE and analyzed by immunoblotting using antibodies against Pcs60p (peroxisomal matrix protein), Cta1p (catalase; peroxisomal matrix protein), Pex11p (peroxisomal membrane protein), Tom40p and Porin (mitochondrial outer membrane protein), Tim23p (mitochondrial inner membrane protein), Kar2p (ER luminal protein), Dpm1p (ER membrane protein). Shown here is the relative concentration of the indicated proteins in each fraction measured by the intensity of the band in the immunoblot analysis. The peroxisomal fractions (3-5) only show very little contamination with mitochondrial and ER membrane markers.

**Supplementary Table 1:** Lipid identifiers of the SwissLipids database

| **feature** | **SwissLipidsName** | **SwissLipidsId** |
| --- | --- | --- |
| CDP DAG_34:1;0 |  |  |
| CDP DAG_34:2;0 |  |  |
| CL 61:1;0 | a cardiolipin |  |
| CL 64:4;0 | a cardiolipin |  |
| CL 66:4;0 | a cardiolipin |  |
| CL 66:5;0 | a cardiolipin |  |
| CL 68:4;0 | a cardiolipin |  |
| CL 68:5;0 | a cardiolipin |  |
| CL 68:6;0 | a cardiolipin |  |
| CL 70:4;0 | a cardiolipin |  |
| CL 70:5;0 | a cardiolipin |  |
| CL 70:6;0 | a cardiolipin |  |
| CL 70:7;0 | a cardiolipin |  |
| CL 72:4;0 | a cardiolipin |  |
| CL 72:5;0 | a cardiolipin |  |
| CL 72:6;0 | a cardiolipin |  |
| CL 72:7;0 | a cardiolipin |  |
| CL 72:8;0 | a cardiolipin |  |
| Cer 32:0;2 | Ceramide (d32:0) | SLM:000391219 |
| Cer 32:1;2 | Ceramide (d32:1) | SLM:000391217 |
| Cer 32:1;3 | Ceramide (t32:1) | SLM:000391227 |
| Cer 32:1;4 | Ceramide (q32:1) |  |
| Cer 32:1;5 | Ceramide (Number of Oh not found for 532:1) |  |
| Cer 34:0;2 | Ceramide (d34:0) | SLM:000391238 |
| Cer 34:0;3 | Ceramide (t34:0) | SLM:000391253 |
| Cer 34:0;4 | Ceramide (q34:0) |  |
| Cer 34:1;2 | Ceramide (d34:1) | SLM:000391236 |
| Cer 34:1;3 | Ceramide (t34:1) | SLM:000391250 |
| Cer 34:1;4 | Ceramide (q34:1) |  |
| Cer 34:1;5 | Ceramide (Number of Oh not found for 534:1) |  |
| Cer 36:0;2 | Ceramide (d36:0) | SLM:000391263 |
| Cer 36:0;3 | Ceramide (t36:0) | SLM:000391280 |
| Cer 36:0;4 | Ceramide (q36:0) |  |
| Cer 36:1;2 | Ceramide (d36:1) | SLM:000391261 |
| Cer 36:1;3 | Ceramide (t36:1) | SLM:000391277 |
| Cer 36:1;4 | Ceramide (q36:1) |  |
| Cer 36:1;5 | Ceramide (Number of Oh not found for 536:1) |  |
| Cer 38:0;2 | Ceramide (d38:0) | SLM:000391292 |
| Cer 38:0;3 | Ceramide (t38:0) | SLM:000391309 |
| Cer 38:0;4 | Ceramide (q38:0) |  |
| Cer 38:1;2 | Ceramide (d38:1) | SLM:000391289 |
| Cer 38:1;3 | Ceramide (t38:1) | SLM:000391306 |
| Cer 38:1;4 | Ceramide (q38:1) |  |
| Cer 38:1;5 | Ceramide (Number of Oh not found for 538:1) |  |
| Cer 40:0;2 | Ceramide (d40:0) | SLM:000391322 |
| Cer 40:0;3 | Ceramide (t40:0) | SLM:000391338 |
| Cer 40:0;4 | Ceramide (q40:0) |  |
| Cer 40:1;2 | Ceramide (d40:1) | SLM:000391319 |
| Cer 40:1;3 | Ceramide (t40:1) | SLM:000391335 |
| Cer 40:1;4 | Ceramide (q40:1) |  |
| Cer 42:0;2 | Ceramide (d42:0) | SLM:000391349 |
| Cer 42:0;3 | Ceramide (t42:0) | SLM:000391365 |
| Cer 42:0;4 | Ceramide (q42:0) |  |
| Cer 42:0;5 | Ceramide (Number of Oh not found for 542:0) |  |
| Cer 42:1;2 | Ceramide (d42:1) | SLM:000391346 |
| Cer 42:1;3 | Ceramide (t42:1) | SLM:000391362 |
| Cer 42:1;4 | Ceramide (q42:1) |  |
| Cer 44:0;2 | Ceramide (d44:0) | SLM:000391375 |
| Cer 44:0;3 | Ceramide (t44:0) | SLM:000391388 |
| Cer 44:0;4 | Ceramide (q44:0) |  |
| Cer 44:0;5 | Ceramide (Number of Oh not found for 544:0) |  |
| Cer 44:1;3 | Ceramide (t44:1) | SLM:000391385 |
| Cer 44:1;4 | Ceramide (q44:1) |  |
| Cer 44:1;5 | Ceramide (Number of Oh not found for 544:1) |  |
| Cer 46:0;3 | Ceramide (t46:0) | SLM:000391410 |
| Cer 46:0;4 | Ceramide (q46:0) |  |
| Cer 46:1;4 | Ceramide (q46:1) |  |
| DAG 14:0;0_15:0;0 | Diacylglycerol (14:0_15:0) | SLM:000308754 |
| DAG 14:0;0_16:0;0 | Diacylglycerol (14:0_16:0) | SLM:000308755 |
| DAG 15:0;0_15:0;0 | Diacylglycerol (15:0_15:0) | SLM:000308823 |
| DAG 12:0;0_18:1;0 | Diacylglycerol (12:0_18:1) | SLM:000308687 |
| DAG 14:0;0_16:1;0 | Diacylglycerol (14:0_16:1) | SLM:000308756 |
| DAG 14:1;0_16:0;0 | Diacylglycerol (14:1_16:0) | SLM:000308790 |
| DAG 14:0;0_17:0;0 | Diacylglycerol (14:0_17:0) | SLM:000308758 |
| DAG 15:0;0_16:0;0 | Diacylglycerol (15:0_16:0) | SLM:000308824 |
| DAG 14:0;0_17:1;0 | Diacylglycerol (14:0_17:1) |  |
| DAG 15:0;0_16:1;0 | Diacylglycerol (15:0_16:1) | SLM:000308825 |
| DAG 15:1;0_16:0;0 | Diacylglycerol (15:1_16:0) |  |
| DAG 15:1;0_16:1;0 | Diacylglycerol (15:1_16:1) |  |
| DAG 14:0;0_18:0;0 | Diacylglycerol (14:0_18:0) | SLM:000308759 |
| DAG 15:0;0_17:0;0 | Diacylglycerol (15:0_17:0) | SLM:000308827 |
| DAG 16:0;0_16:0;0 | Diacylglycerol (16:0_16:0) | SLM:000308857 |
| DAG 14:0;0_18:1;0 | Diacylglycerol (14:0_18:1) | SLM:000308760 |
| DAG 14:1;0_18:0;0 | Diacylglycerol (14:1_18:0) | SLM:000308794 |
| DAG 15:0;0_17:1;0 | Diacylglycerol (15:0_17:1) |  |
| DAG 16:1;0_16:0;0 | Diacylglycerol (16:1_16:0) |  |
| DAG 14:0;0_18:2;0 | Diacylglycerol (14:0_18:2) | SLM:000308761 |
| DAG 14:1;0_18:1;0 | Diacylglycerol (14:1_18:1) | SLM:000308795 |
| DAG 16:1;0_16:1;0 | Diacylglycerol (16:1_16:1) | SLM:000308890 |
| DAG 16:2;0_16:0;0 | Diacylglycerol (16:2_16:0) |  |
| DAG 14:0;0_18:3;0 | Diacylglycerol (14:0_18:3) | SLM:000308762 |
| DAG 14:1;0_18:2;0 | Diacylglycerol (14:1_18:2) | SLM:000308796 |
| DAG 16:2;0_16:1;0 | Diacylglycerol (16:2_16:1) |  |
| DAG 15:0;0_18:0;0 | Diacylglycerol (15:0_18:0) | SLM:000308828 |
| DAG 16:0;0_17:0;0 | Diacylglycerol (16:0_17:0) | SLM:000308860 |
| DAG 15:0;0_18:1;0 | Diacylglycerol (15:0_18:1) | SLM:000308829 |
| DAG 16:0;0_17:1;0 | Diacylglycerol (16:0_17:1) |  |
| DAG 16:1;0_17:0;0 | Diacylglycerol (16:1_17:0) | SLM:000308892 |
| DAG 15:0;0_18:2;0 | Diacylglycerol (15:0_18:2) | SLM:000308830 |
| DAG 15:1;0_18:1;0 | Diacylglycerol (15:1_18:1) |  |
| DAG 16:0;0_17:2;0 | Diacylglycerol (16:0_17:2) |  |
| DAG 16:1;0_17:1;0 | Diacylglycerol (16:1_17:1) |  |
| DAG 15:1;0_18:2;0 | Diacylglycerol (15:1_18:2) |  |
| DAG 16:0;0_18:0;0 | Diacylglycerol (16:0_18:0) | SLM:000308861 |
| DAG 16:0;0_18:1;0 | Diacylglycerol (16:0_18:1) | SLM:000308862 |
| DAG 16:0;0_18:2;0 | Diacylglycerol (16:0_18:2) | SLM:000308863 |
| DAG 16:1;0_18:1;0 | Diacylglycerol (16:1_18:1) | SLM:000308894 |
| DAG 17:1;0_17:1;0 | Diacylglycerol (17:1_17:1) |  |
| DAG 16:0;0_18:3;0 | Diacylglycerol (16:0_18:3) | SLM:000308864 |
| DAG 16:1;0_18:2;0 | Diacylglycerol (16:1_18:2) | SLM:000308895 |
| DAG 16:2;0_18:1;0 | Diacylglycerol (16:2_18:1) | SLM:000308925 |
| DAG 16:1;0_18:3;0 | Diacylglycerol (16:1_18:3) | SLM:000308896 |
| DAG 16:2;0_18:2;0 | Diacylglycerol (16:2_18:2) | SLM:000308926 |
| DAG 16:0;0_19:1;0 | Diacylglycerol (16:0_19:1) |  |
| DAG 17:0;0_18:1;0 | Diacylglycerol (17:0_18:1) | SLM:000308955 |
| DAG 17:1;0_18:0;0 | Diacylglycerol (17:1_18:0) |  |
| DAG 17:0;0_18:2;0 | Diacylglycerol (17:0_18:2) | SLM:000308956 |
| DAG 17:1;0_18:1;0 | Diacylglycerol (17:1_18:1) |  |
| DAG 17:1;0_18:2;0 | Diacylglycerol (17:1_18:2) |  |
| DAG 17:2;0_18:1;0 | Diacylglycerol (17:2_18:1) |  |
| DAG 18:0;0_18:0;0 | Diacylglycerol (18:0_18:0) | SLM:000308983 |
| DAG 16:0;0_20:1;0 | Diacylglycerol (16:0_20:1) | SLM:000308868 |
| DAG 18:1;0_18:0;0 | Diacylglycerol (18:1_18:0) |  |
| DAG 16:1;0_20:1;0 | Diacylglycerol (16:1_20:1) | SLM:000308900 |
| DAG 18:1;0_18:1;0 | Diacylglycerol (18:1_18:1) | SLM:000309012 |
| DAG 18:2;0_18:0;0 | Diacylglycerol (18:2_18:0) |  |
| DAG 16:1;0_20:2;0 | Diacylglycerol (16:1_20:2) | SLM:000308901 |
| DAG 18:2;0_18:1;0 | Diacylglycerol (18:2_18:1) |  |
| DAG 18:3;0_18:0;0 | Diacylglycerol (18:3_18:0) |  |
| DAG 18:2;0_18:2;0 | Diacylglycerol (18:2_18:2) | SLM:000309040 |
| DAG 18:3;0_18:1;0 | Diacylglycerol (18:3_18:1) |  |
| DAG 18:1;0_19:0;0 | Diacylglycerol (18:1_19:0) | SLM:000309016 |
| DAG 18:1;0_19:1;0 | Diacylglycerol (18:1_19:1) |  |
| DAG 18:1;0_20:0;0 | Diacylglycerol (18:1_20:0) | SLM:000309017 |
| DAG 18:1;0_20:1;0 | Diacylglycerol (18:1_20:1) | SLM:000309018 |
| DAG 20:0;0_20:0;0 | Diacylglycerol (20:0_20:0) | SLM:000309142 |
| EE 28:3;0_16:1;0 |  |  |
| EE 28:3;0_18:1;0 |  |  |
| EE 28:3;0_18:2;0 |  |  |
| IPC 38:0;3 |  |  |
| IPC 38:1;2 |  |  |
| IPC 38:1;3 |  |  |
| IPC 40:0;3 |  |  |
| IPC 40:0;4 |  |  |
| IPC 40:1;3 |  |  |
| IPC 40:1;4 |  |  |
| IPC 42:0;3 |  |  |
| IPC 42:0;4 |  |  |
| IPC 42:0;5 |  |  |
| IPC 42:1;3 |  |  |
| IPC 42:1;4 |  |  |
| IPC 42:1;5 |  |  |
| IPC 44:0;3 |  |  |
| IPC 44:0;4 |  |  |
| IPC 44:0;5 |  |  |
| IPC 44:1;3 |  |  |
| IPC 44:1;4 |  |  |
| IPC 44:1;5 |  |  |
| IPC 46:0;3 |  |  |
| IPC 46:0;4 |  |  |
| IPC 46:0;5 |  |  |
| IPC 46:1;3 |  |  |
| IPC 46:1;4 |  |  |
| LPA 16:0;0 | Phosphatidate (16:0_0:0) | SLM:000059759 |
| LPA 16:1;0 | Phosphatidate (16:1_0:0) | SLM:000059813 |
| LPA 16:2;0 | Phosphatidate (16:2_0:0) | SLM:000059866 |
| LPA 18:0;0 | Phosphatidate (18:0_0:0) | SLM:000059969 |
| LPA 18:1;0 | Phosphatidate (18:1_0:0) | SLM:000060019 |
| LPA 18:2;0 | Phosphatidate (18:2_0:0) | SLM:000060068 |
| LPA 18:3;0 | Phosphatidate (18:3_0:0) | SLM:000060116 |
| LPA 20:0;0 | Phosphatidate (20:0_0:0) | SLM:000060254 |
| LPC 10:0;0 | Phosphatidylcholine (10:0_0:0) | SLM:000063378 |
| LPC 12:1;0 | Phosphatidylcholine (12:1_0:0) |  |
| LPC 14:0;0 | Phosphatidylcholine (14:0_0:0) | SLM:000063555 |
| LPC 14:1;0 | Phosphatidylcholine (14:1_0:0) | SLM:000063612 |
| LPC 16:0;0 | Phosphatidylcholine (16:0_0:0) | SLM:000063723 |
| LPC 16:1;0 | Phosphatidylcholine (16:1_0:0) | SLM:000063777 |
| LPC 18:0;0 | Phosphatidylcholine (18:0_0:0) | SLM:000063933 |
| LPC 18:1;0 | Phosphatidylcholine (18:1_0:0) | SLM:000063983 |
| LPC 18:2;0 | Phosphatidylcholine (18:2_0:0) | SLM:000064032 |
| LPC 18:3;0 | Phosphatidylcholine (18:3_0:0) | SLM:000064080 |
| LPC 20:1;0 | Phosphatidylcholine (20:1_0:0) | SLM:000064262 |
| LPE 14:0;0 | Phosphatidylethanolamine (14:0_0:0) | SLM:000067519 |
| LPE 16:0;0 | Phosphatidylethanolamine (16:0_0:0) | SLM:000067687 |
| LPE 16:1;0 | Phosphatidylethanolamine (16:1_0:0) | SLM:000067741 |
| LPE 16:2;0 | Phosphatidylethanolamine (16:2_0:0) | SLM:000067794 |
| LPE 18:0;0 | Phosphatidylethanolamine (18:0_0:0) | SLM:000067897 |
| LPE 18:1;0 | Phosphatidylethanolamine (18:1_0:0) | SLM:000067947 |
| LPE 18:2;0 | Phosphatidylethanolamine (18:2_0:0) | SLM:000067996 |
| LPE 20:1;0 | Phosphatidylethanolamine (20:1_0:0) | SLM:000068226 |
| LPE 20:2;0 | Phosphatidylethanolamine (20:2_0:0) | SLM:000068269 |
| LPE 24:0;0 | Phosphatidylethanolamine (24:0_0:0) | SLM:000068707 |
| LPI 14:0;0 | Phosphatidylinositol (14:0_0:0) | SLM:000073627 |
| LPI 16:0;0 | Phosphatidylinositol (16:0_0:0) | SLM:000073795 |
| LPI 16:1;0 | Phosphatidylinositol (16:1_0:0) | SLM:000073849 |
| LPI 18:0;0 | Phosphatidylinositol (18:0_0:0) | SLM:000074005 |
| LPI 18:1;0 | Phosphatidylinositol (18:1_0:0) | SLM:000074055 |
| LPI 18:2;0 | Phosphatidylinositol (18:2_0:0) | SLM:000074104 |
| LPI 18:3;0 | Phosphatidylinositol (18:3_0:0) | SLM:000074152 |
| LPI 20:1;0 | Phosphatidylinositol (20:1_0:0) | SLM:000074334 |
| LPS 14:0;0 | Phosphatidylserine (14:0_0:0) | SLM:000077591 |
| LPS 14:1;0 | Phosphatidylserine (14:1_0:0) | SLM:000077648 |
| LPS 16:0;0 | Phosphatidylserine (16:0_0:0) | SLM:000077759 |
| LPS 16:1;0 | Phosphatidylserine (16:1_0:0) | SLM:000077813 |
| LPS 18:1;0 | Phosphatidylserine (18:1_0:0) | SLM:000078019 |
| LPS 18:2;0 | Phosphatidylserine (18:2_0:0) | SLM:000078068 |
| M(IP)2C 42:0;3 |  |  |
| M(IP)2C 42:0;4 |  |  |
| M(IP)2C 42:1;4 |  |  |
| M(IP)2C 44:0;3 |  |  |
| M(IP)2C 44:0;4 |  |  |
| M(IP)2C 44:0;5 |  |  |
| M(IP)2C 44:1;2 |  |  |
| M(IP)2C 44:1;3 |  |  |
| M(IP)2C 44:1;4 |  |  |
| M(IP)2C 44:1;5 |  |  |
| M(IP)2C 46:0;3 |  |  |
| M(IP)2C 46:0;4 |  |  |
| M(IP)2C 46:1;4 |  |  |
| MIPC 42:0;4 |  |  |
| MIPC 44:0;3 |  |  |
| MIPC 44:0;4 |  |  |
| MIPC 44:0;5 |  |  |
| MIPC 44:1;4 |  |  |
| MIPC 46:0;4 |  |  |
| PA 16:0;0_14:1;0 | Phosphatidate (16:0_14:1) |  |
| PA 16:1;0_14:0;0 | Phosphatidate (16:1_14:0) |  |
| PA 16:1;0_15:0;0 | Phosphatidate (16:1_15:0) |  |
| PA 17:1;0_14:0;0 | Phosphatidate (17:1_14:0) |  |
| PA 16:0;0_16:1;0 | Phosphatidate (16:0_16:1) | SLM:000059761 |
| PA 17:1;0_15:0;0 | Phosphatidate (17:1_15:0) |  |
| PA 18:1;0_14:0;0 | Phosphatidate (18:1_14:0) |  |
| PA 16:1;0_16:1;0 | Phosphatidate (16:1_16:1) | SLM:000059814 |
| PA 18:1;0_14:1;0 | Phosphatidate (18:1_14:1) |  |
| PA 18:2;0_14:0;0 | Phosphatidate (18:2_14:0) |  |
| PA 17:0;0_16:1;0 | Phosphatidate (17:0_16:1) |  |
| PA 17:1;0_16:0;0 | Phosphatidate (17:1_16:0) |  |
| PA 18:1;0_15:0;0 | Phosphatidate (18:1_15:0) |  |
| PA 19:1;0_14:0;0 | Phosphatidate (19:1_14:0) |  |
| PA 17:1;0_16:1;0 | Phosphatidate (17:1_16:1) |  |
| PA 18:1;0_15:1;0 | Phosphatidate (18:1_15:1) |  |
| PA 18:2;0_15:0;0 | Phosphatidate (18:2_15:0) |  |
| PA 17:0;0_17:0;0 | Phosphatidate (17:0_17:0) | SLM:000059919 |
| PA 18:0;0_16:0;0 | Phosphatidate (18:0_16:0) |  |
| PA 17:5;0_17:5;0 | Phosphatidate (17:5_17:5) |  |
| PA 17:0;0_17:1;0 | Phosphatidate (17:0_17:1) |  |
| PA 18:0;0_16:1;0 | Phosphatidate (18:0_16:1) |  |
| PA 18:1;0_16:0;0 | Phosphatidate (18:1_16:0) |  |
| PA 17:1;0_17:1;0 | Phosphatidate (17:1_17:1) |  |
| PA 18:1;0_16:1;0 | Phosphatidate (18:1_16:1) |  |
| PA 18:2;0_16:0;0 | Phosphatidate (18:2_16:0) |  |
| PA 20:1;0_14:1;0 | Phosphatidate (20:1_14:1) |  |
| PA 18:1;0_16:2;0 | Phosphatidate (18:1_16:2) |  |
| PA 18:2;0_16:1;0 | Phosphatidate (18:2_16:1) |  |
| PA 18:3;0_16:1;0 | Phosphatidate (18:3_16:1) |  |
| PA 18:0;0_17:0;0 | Phosphatidate (18:0_17:0) |  |
| PA 18:0;0_17:1;0 | Phosphatidate (18:0_17:1) |  |
| PA 18:1;0_17:0;0 | Phosphatidate (18:1_17:0) |  |
| PA 18:1;0_17:1;0 | Phosphatidate (18:1_17:1) |  |
| PA 18:2;0_17:0;0 | Phosphatidate (18:2_17:0) |  |
| PA 18:0;0_18:1;0 | Phosphatidate (18:0_18:1) | SLM:000059971 |
| PA 18:0;0_18:2;0 | Phosphatidate (18:0_18:2) | SLM:000059972 |
| PA 18:1;0_18:1;0 | Phosphatidate (18:1_18:1) | SLM:000060020 |
| PA 20:1;0_16:1;0 | Phosphatidate (20:1_16:1) |  |
| PA 18:0;0_18:3;0 | Phosphatidate (18:0_18:3) | SLM:000059973 |
| PA 18:1;0_18:2;0 | Phosphatidate (18:1_18:2) | SLM:000060021 |
| PA 18:1;0_18:3;0 | Phosphatidate (18:1_18:3) | SLM:000060022 |
| PA 18:2;0_18:2;0 | Phosphatidate (18:2_18:2) | SLM:000060069 |
| PA 20:1;0_18:1;0 | Phosphatidate (20:1_18:1) |  |
| PC 14:1;0_16:1;0 | Phosphatidylcholine (14:1_16:1) | SLM:000063616 |
| PC 14:0;0_17:1;0 | Phosphatidylcholine (14:0_17:1) |  |
| PC 15:0;0_16:1;0 | Phosphatidylcholine (15:0_16:1) | SLM:000063671 |
| PC 15:1;0_16:0;0 | Phosphatidylcholine (15:1_16:0) |  |
| PC 15:1;0_16:1;0 | Phosphatidylcholine (15:1_16:1) |  |
| PC 14:0;0_18:1;0 | Phosphatidylcholine (14:0_18:1) | SLM:000063564 |
| PC 14:1;0_18:0;0 | Phosphatidylcholine (14:1_18:0) | SLM:000063619 |
| PC 15:0;0_17:1;0 | Phosphatidylcholine (15:0_17:1) |  |
| PC 16:1;0_16:0;0 | Phosphatidylcholine (16:1_16:0) |  |
| PC 14:0;0_18:2;0 | Phosphatidylcholine (14:0_18:2) | SLM:000063565 |
| PC 14:1;0_18:1;0 | Phosphatidylcholine (14:1_18:1) | SLM:000063620 |
| PC 16:1;0_16:1;0 | Phosphatidylcholine (16:1_16:1) | SLM:000063778 |
| PC 16:2;0_16:0;0 | Phosphatidylcholine (16:2_16:0) |  |
| PC 14:0;0_18:3;0 | Phosphatidylcholine (14:0_18:3) | SLM:000063566 |
| PC 14:1;0_18:2;0 | Phosphatidylcholine (14:1_18:2) | SLM:000063621 |
| PC 16:2;0_16:1;0 | Phosphatidylcholine (16:2_16:1) |  |
| PC 15:0;0_18:1;0 | Phosphatidylcholine (15:0_18:1) | SLM:000063675 |
| PC 16:0;0_17:1;0 | Phosphatidylcholine (16:0_17:1) |  |
| PC 16:1;0_17:0;0 | Phosphatidylcholine (16:1_17:0) | SLM:000063780 |
| PC 15:0;0_18:2;0 | Phosphatidylcholine (15:0_18:2) | SLM:000063676 |
| PC 15:1;0_18:1;0 | Phosphatidylcholine (15:1_18:1) |  |
| PC 16:1;0_17:1;0 | Phosphatidylcholine (16:1_17:1) |  |
| PC 15:1;0_18:2;0 | Phosphatidylcholine (15:1_18:2) |  |
| PC 16:1;0_17:2;0 | Phosphatidylcholine (16:1_17:2) |  |
| PC 16:2;0_17:1;0 | Phosphatidylcholine (16:2_17:1) |  |
| PC 16:0;0_18:0;0 | Phosphatidylcholine (16:0_18:0) | SLM:000063728 |
| PC 17:0;0_17:0;0 | Phosphatidylcholine (17:0_17:0) | SLM:000063883 |
| PC 16:0;0_18:1;0 | Phosphatidylcholine (16:0_18:1) | SLM:000063729 |
| PC 17:1;0_17:0;0 | Phosphatidylcholine (17:1_17:0) |  |
| PC 16:0;0_18:2;0 | Phosphatidylcholine (16:0_18:2) | SLM:000063730 |
| PC 16:1;0_18:1;0 | Phosphatidylcholine (16:1_18:1) | SLM:000063782 |
| PC 17:1;0_17:1;0 | Phosphatidylcholine (17:1_17:1) |  |
| PC 16:0;0_18:3;0 | Phosphatidylcholine (16:0_18:3) | SLM:000063731 |
| PC 16:1;0_18:2;0 | Phosphatidylcholine (16:1_18:2) | SLM:000063783 |
| PC 16:2;0_18:1;0 | Phosphatidylcholine (16:2_18:1) | SLM:000063834 |
| PC 16:1;0_18:3;0 | Phosphatidylcholine (16:1_18:3) | SLM:000063784 |
| PC 16:2;0_18:2;0 | Phosphatidylcholine (16:2_18:2) | SLM:000063835 |
| PC 17:0;0_18:1;0 | Phosphatidylcholine (17:0_18:1) | SLM:000063885 |
| PC 16:1;0_19:1;0 | Phosphatidylcholine (16:1_19:1) |  |
| PC 17:0;0_18:2;0 | Phosphatidylcholine (17:0_18:2) | SLM:000063886 |
| PC 17:1;0_18:1;0 | Phosphatidylcholine (17:1_18:1) |  |
| PC 17:1;0_18:2;0 | Phosphatidylcholine (17:1_18:2) |  |
| PC 17:2;0_18:1;0 | Phosphatidylcholine (17:2_18:1) |  |
| PC 17:1;0_18:3;0 | Phosphatidylcholine (17:1_18:3) |  |
| PC 16:1;0_20:1;0 | Phosphatidylcholine (16:1_20:1) | SLM:000063788 |
| PC 18:1;0_18:1;0 | Phosphatidylcholine (18:1_18:1) | SLM:000063984 |
| PC 18:2;0_18:0;0 | Phosphatidylcholine (18:2_18:0) |  |
| PC 16:1;0_20:2;0 | Phosphatidylcholine (16:1_20:2) | SLM:000063789 |
| PC 18:2;0_18:1;0 | Phosphatidylcholine (18:2_18:1) |  |
| PC 18:3;0_18:0;0 | Phosphatidylcholine (18:3_18:0) |  |
| PC 16:0;0_20:4;0 | Phosphatidylcholine (16:0_20:4) | SLM:000063738 |
| PC 18:2;0_18:2;0 | Phosphatidylcholine (18:2_18:2) | SLM:000064033 |
| PC 18:3;0_18:1;0 | Phosphatidylcholine (18:3_18:1) |  |
| PC 18:3;0_18:2;0 | Phosphatidylcholine (18:3_18:2) |  |
| PC 17:1;0_20:1;0 | Phosphatidylcholine (17:1_20:1) |  |
| PC 18:1;0_19:1;0 | Phosphatidylcholine (18:1_19:1) |  |
| PC 16:1;0_22:1;0 | Phosphatidylcholine (16:1_22:1) | SLM:000063795 |
| PC 18:1;0_20:1;0 | Phosphatidylcholine (18:1_20:1) | SLM:000063990 |
| PC 18:1;0_20:2;0 | Phosphatidylcholine (18:1_20:2) | SLM:000063991 |
| PC 18:2;0_20:1;0 | Phosphatidylcholine (18:2_20:1) | SLM:000064038 |
| PE 15:0;0_14:0;0 | Phosphatidylethanolamine (15:0_14:0) |  |
| PE 16:0;0_14:0;0 | Phosphatidylethanolamine (16:0_14:0) |  |
| PE 16:0;0_14:1;0 | Phosphatidylethanolamine (16:0_14:1) |  |
| PE 16:1;0_14:0;0 | Phosphatidylethanolamine (16:1_14:0) |  |
| PE 17:0;0_13:1;0 | Phosphatidylethanolamine (17:0_13:1) |  |
| PE 16:1;0_14:1;0 | Phosphatidylethanolamine (16:1_14:1) |  |
| PE 16:0;0_15:1;0 | Phosphatidylethanolamine (16:0_15:1) |  |
| PE 16:1;0_15:0;0 | Phosphatidylethanolamine (16:1_15:0) |  |
| PE 17:1;0_14:0;0 | Phosphatidylethanolamine (17:1_14:0) |  |
| PE 16:1;0_15:1;0 | Phosphatidylethanolamine (16:1_15:1) |  |
| PE 16:0;0_16:1;0 | Phosphatidylethanolamine (16:0_16:1) | SLM:000067689 |
| PE 17:1;0_15:0;0 | Phosphatidylethanolamine (17:1_15:0) |  |
| PE 18:0;0_14:1;0 | Phosphatidylethanolamine (18:0_14:1) |  |
| PE 18:1;0_14:0;0 | Phosphatidylethanolamine (18:1_14:0) |  |
| PE 16:0;0_16:2;0 | Phosphatidylethanolamine (16:0_16:2) | SLM:000067690 |
| PE 16:1;0_16:1;0 | Phosphatidylethanolamine (16:1_16:1) | SLM:000067742 |
| PE 18:1;0_14:1;0 | Phosphatidylethanolamine (18:1_14:1) |  |
| PE 18:2;0_14:0;0 | Phosphatidylethanolamine (18:2_14:0) |  |
| PE 16:1;0_16:2;0 | Phosphatidylethanolamine (16:1_16:2) | SLM:000067743 |
| PE 18:2;0_14:1;0 | Phosphatidylethanolamine (18:2_14:1) |  |
| PE 18:3;0_14:0;0 | Phosphatidylethanolamine (18:3_14:0) |  |
| PE 17:0;0_16:0;0 | Phosphatidylethanolamine (17:0_16:0) |  |
| PE 17:1;0_16:0;0 | Phosphatidylethanolamine (17:1_16:0) |  |
| PE 18:1;0_15:0;0 | Phosphatidylethanolamine (18:1_15:0) |  |
| PE 17:1;0_16:1;0 | Phosphatidylethanolamine (17:1_16:1) |  |
| PE 17:2;0_16:0;0 | Phosphatidylethanolamine (17:2_16:0) |  |
| PE 18:1;0_15:1;0 | Phosphatidylethanolamine (18:1_15:1) |  |
| PE 18:2;0_15:0;0 | Phosphatidylethanolamine (18:2_15:0) |  |
| PE 18:1;0_16:0;0 | Phosphatidylethanolamine (18:1_16:0) |  |
| PE 17:1;0_17:1;0 | Phosphatidylethanolamine (17:1_17:1) |  |
| PE 18:1;0_16:1;0 | Phosphatidylethanolamine (18:1_16:1) |  |
| PE 18:2;0_16:0;0 | Phosphatidylethanolamine (18:2_16:0) |  |
| PE 18:1;0_16:2;0 | Phosphatidylethanolamine (18:1_16:2) |  |
| PE 18:2;0_16:1;0 | Phosphatidylethanolamine (18:2_16:1) |  |
| PE 18:3;0_16:0;0 | Phosphatidylethanolamine (18:3_16:0) |  |
| PE 18:2;0_16:2;0 | Phosphatidylethanolamine (18:2_16:2) |  |
| PE 18:3;0_16:1;0 | Phosphatidylethanolamine (18:3_16:1) |  |
| PE 18:1;0_17:1;0 | Phosphatidylethanolamine (18:1_17:1) |  |
| PE 18:2;0_17:0;0 | Phosphatidylethanolamine (18:2_17:0) |  |
| PE 19:1;0_16:1;0 | Phosphatidylethanolamine (19:1_16:1) |  |
| PE 18:1;0_17:2;0 | Phosphatidylethanolamine (18:1_17:2) |  |
| PE 18:2;0_17:1;0 | Phosphatidylethanolamine (18:2_17:1) |  |
| PE 18:0;0_18:2;0 | Phosphatidylethanolamine (18:0_18:2) | SLM:000067900 |
| PE 18:1;0_18:1;0 | Phosphatidylethanolamine (18:1_18:1) | SLM:000067948 |
| PE 20:1;0_16:1;0 | Phosphatidylethanolamine (20:1_16:1) |  |
| PE 18:0;0_18:3;0 | Phosphatidylethanolamine (18:0_18:3) | SLM:000067901 |
| PE 18:1;0_18:2;0 | Phosphatidylethanolamine (18:1_18:2) | SLM:000067949 |
| PE 20:2;0_16:1;0 | Phosphatidylethanolamine (20:2_16:1) |  |
| PE 18:1;0_18:3;0 | Phosphatidylethanolamine (18:1_18:3) | SLM:000067950 |
| PE 18:2;0_18:2;0 | Phosphatidylethanolamine (18:2_18:2) | SLM:000067997 |
| PE 18:2;0_18:3;0 | Phosphatidylethanolamine (18:2_18:3) | SLM:000067998 |
| PE 19:1;0_18:1;0 | Phosphatidylethanolamine (19:1_18:1) |  |
| PE 20:1;0_17:1;0 | Phosphatidylethanolamine (20:1_17:1) |  |
| PE 19:1;0_18:2;0 | Phosphatidylethanolamine (19:1_18:2) |  |
| PE 19:1;0_19:1;0 | Phosphatidylethanolamine (19:1_19:1) |  |
| PE 20:1;0_18:1;0 | Phosphatidylethanolamine (20:1_18:1) |  |
| PE 20:1;0_18:2;0 | Phosphatidylethanolamine (20:1_18:2) |  |
| PE 20:2;0_18:1;0 | Phosphatidylethanolamine (20:2_18:1) |  |
| PG 12:1;0_18:0;0 | Phosphatidylglycerol (12:1_18:0) |  |
| PG 13:1;0_17:0;0 | Phosphatidylglycerol (13:1_17:0) |  |
| PG 14:0;0_16:1;0 | Phosphatidylglycerol (14:0_16:1) | SLM:000071488 |
| PG 14:1;0_16:0;0 | Phosphatidylglycerol (14:1_16:0) | SLM:000071543 |
| PG 14:0;0_17:1;0 | Phosphatidylglycerol (14:0_17:1) |  |
| PG 15:0;0_16:1;0 | Phosphatidylglycerol (15:0_16:1) | SLM:000071599 |
| PG 14:0;0_18:1;0 | Phosphatidylglycerol (14:0_18:1) | SLM:000071492 |
| PG 14:1;0_18:0;0 | Phosphatidylglycerol (14:1_18:0) | SLM:000071547 |
| PG 15:0;0_17:1;0 | Phosphatidylglycerol (15:0_17:1) |  |
| PG 16:1;0_16:0;0 | Phosphatidylglycerol (16:1_16:0) |  |
| PG 14:0;0_18:2;0 | Phosphatidylglycerol (14:0_18:2) | SLM:000071493 |
| PG 16:1;0_16:1;0 | Phosphatidylglycerol (16:1_16:1) | SLM:000071706 |
| PG 15:0;0_18:1;0 | Phosphatidylglycerol (15:0_18:1) | SLM:000071603 |
| PG 16:0;0_17:1;0 | Phosphatidylglycerol (16:0_17:1) |  |
| PG 16:1;0_17:0;0 | Phosphatidylglycerol (16:1_17:0) | SLM:000071708 |
| PG 15:0;0_18:2;0 | Phosphatidylglycerol (15:0_18:2) | SLM:000071604 |
| PG 16:1;0_17:1;0 | Phosphatidylglycerol (16:1_17:1) |  |
| PG 16:0;0_18:1;0 | Phosphatidylglycerol (16:0_18:1) | SLM:000071657 |
| PG 16:1;0_18:0;0 | Phosphatidylglycerol (16:1_18:0) | SLM:000071709 |
| PG 17:1;0_17:0;0 | Phosphatidylglycerol (17:1_17:0) |  |
| PG 16:0;0_18:2;0 | Phosphatidylglycerol (16:0_18:2) | SLM:000071658 |
| PG 16:1;0_18:1;0 | Phosphatidylglycerol (16:1_18:1) | SLM:000071710 |
| PG 17:1;0_17:1;0 | Phosphatidylglycerol (17:1_17:1) |  |
| PG 16:0;0_18:3;0 | Phosphatidylglycerol (16:0_18:3) | SLM:000071659 |
| PG 16:1;0_18:2;0 | Phosphatidylglycerol (16:1_18:2) | SLM:000071711 |
| PG 17:0;0_18:1;0 | Phosphatidylglycerol (17:0_18:1) | SLM:000071813 |
| PG 17:1;0_18:0;0 | Phosphatidylglycerol (17:1_18:0) |  |
| PG 17:0;0_18:2;0 | Phosphatidylglycerol (17:0_18:2) | SLM:000071814 |
| PG 17:1;0_18:1;0 | Phosphatidylglycerol (17:1_18:1) |  |
| PG 18:1;0_18:1;0 | Phosphatidylglycerol (18:1_18:1) | SLM:000071912 |
| PG 18:2;0_18:0;0 | Phosphatidylglycerol (18:2_18:0) |  |
| PG 18:2;0_18:1;0 | Phosphatidylglycerol (18:2_18:1) |  |
| PI 14:0;0_14:0;0 | Phosphatidylinositol (14:0_14:0) | SLM:000073628 |
| PI 16:0;0_12:0;0 | Phosphatidylinositol (16:0_12:0) |  |
| PI 15:0;0_15:0;0 | Phosphatidylinositol (15:0_15:0) | SLM:000073741 |
| PI 16:0;0_14:0;0 | Phosphatidylinositol (16:0_14:0) |  |
| PI 18:0;0_12:0;0 | Phosphatidylinositol (18:0_12:0) |  |
| PI 16:0;0_14:1;0 | Phosphatidylinositol (16:0_14:1) |  |
| PI 16:1;0_14:0;0 | Phosphatidylinositol (16:1_14:0) |  |
| PI 18:1;0_12:0;0 | Phosphatidylinositol (18:1_12:0) |  |
| PI 16:0;0_15:1;0 | Phosphatidylinositol (16:0_15:1) |  |
| PI 16:1;0_15:0;0 | Phosphatidylinositol (16:1_15:0) |  |
| PI 17:1;0_14:0;0 | Phosphatidylinositol (17:1_14:0) |  |
| PI 16:0;0_16:1;0 | Phosphatidylinositol (16:0_16:1) | SLM:000073797 |
| PI 17:1;0_15:0;0 | Phosphatidylinositol (17:1_15:0) |  |
| PI 18:0;0_14:1;0 | Phosphatidylinositol (18:0_14:1) |  |
| PI 18:1;0_14:0;0 | Phosphatidylinositol (18:1_14:0) |  |
| PI 16:0;0_16:2;0 | Phosphatidylinositol (16:0_16:2) | SLM:000073798 |
| PI 16:1;0_16:1;0 | Phosphatidylinositol (16:1_16:1) | SLM:000073850 |
| PI 18:1;0_14:1;0 | Phosphatidylinositol (18:1_14:1) |  |
| PI 18:2;0_14:0;0 | Phosphatidylinositol (18:2_14:0) |  |
| PI 16:1;0_16:2;0 | Phosphatidylinositol (16:1_16:2) | SLM:000073851 |
| PI 17:0;0_16:1;0 | Phosphatidylinositol (17:0_16:1) |  |
| PI 17:1;0_16:0;0 | Phosphatidylinositol (17:1_16:0) |  |
| PI 18:1;0_15:0;0 | Phosphatidylinositol (18:1_15:0) |  |
| PI 19:1;0_14:0;0 | Phosphatidylinositol (19:1_14:0) |  |
| PI 17:1;0_16:1;0 | Phosphatidylinositol (17:1_16:1) |  |
| PI 17:2;0_16:0;0 | Phosphatidylinositol (17:2_16:0) |  |
| PI 18:1;0_15:1;0 | Phosphatidylinositol (18:1_15:1) |  |
| PI 18:2;0_15:0;0 | Phosphatidylinositol (18:2_15:0) |  |
| PI 18:0;0_16:1;0 | Phosphatidylinositol (18:0_16:1) |  |
| PI 18:1;0_16:0;0 | Phosphatidylinositol (18:1_16:0) |  |
| PI 17:1;0_17:1;0 | Phosphatidylinositol (17:1_17:1) |  |
| PI 18:1;0_16:1;0 | Phosphatidylinositol (18:1_16:1) |  |
| PI 18:2;0_16:0;0 | Phosphatidylinositol (18:2_16:0) |  |
| PI 18:1;0_16:2;0 | Phosphatidylinositol (18:1_16:2) |  |
| PI 18:2;0_16:1;0 | Phosphatidylinositol (18:2_16:1) |  |
| PI 18:3;0_16:0;0 | Phosphatidylinositol (18:3_16:0) |  |
| PI 18:2;0_16:2;0 | Phosphatidylinositol (18:2_16:2) |  |
| PI 18:3;0_16:1;0 | Phosphatidylinositol (18:3_16:1) |  |
| PI 18:0;0_17:1;0 | Phosphatidylinositol (18:0_17:1) |  |
| PI 18:1;0_17:0;0 | Phosphatidylinositol (18:1_17:0) |  |
| PI 19:1;0_16:0;0 | Phosphatidylinositol (19:1_16:0) |  |
| PI 20:1;0_15:0;0 | Phosphatidylinositol (20:1_15:0) |  |
| PI 18:1;0_17:1;0 | Phosphatidylinositol (18:1_17:1) |  |
| PI 18:2;0_17:0;0 | Phosphatidylinositol (18:2_17:0) |  |
| PI 19:1;0_16:1;0 | Phosphatidylinositol (19:1_16:1) |  |
| PI 18:1;0_17:2;0 | Phosphatidylinositol (18:1_17:2) |  |
| PI 18:2;0_17:1;0 | Phosphatidylinositol (18:2_17:1) |  |
| PI 18:3;0_17:0;0 | Phosphatidylinositol (18:3_17:0) |  |
| PI 18:0;0_18:1;0 | Phosphatidylinositol (18:0_18:1) | SLM:000074007 |
| PI 20:1;0_16:0;0 | Phosphatidylinositol (20:1_16:0) |  |
| PI 18:0;0_18:2;0 | Phosphatidylinositol (18:0_18:2) | SLM:000074008 |
| PI 18:1;0_18:1;0 | Phosphatidylinositol (18:1_18:1) | SLM:000074056 |
| PI 20:1;0_16:1;0 | Phosphatidylinositol (20:1_16:1) |  |
| PI 18:0;0_18:3;0 | Phosphatidylinositol (18:0_18:3) | SLM:000074009 |
| PI 18:1;0_18:2;0 | Phosphatidylinositol (18:1_18:2) | SLM:000074057 |
| PI 19:2;0_17:1;0 | Phosphatidylinositol (19:2_17:1) |  |
| PI 20:2;0_16:1;0 | Phosphatidylinositol (20:2_16:1) |  |
| PI 18:1;0_18:3;0 | Phosphatidylinositol (18:1_18:3) | SLM:000074058 |
| PI 18:2;0_18:2;0 | Phosphatidylinositol (18:2_18:2) | SLM:000074105 |
| PI 18:1;0_18:4;0 | Phosphatidylinositol (18:1_18:4) | SLM:000074059 |
| PI 18:2;0_18:3;0 | Phosphatidylinositol (18:2_18:3) | SLM:000074106 |
| PI 19:5;0_17:0;0 | Phosphatidylinositol (19:5_17:0) |  |
| PI 19:1;0_18:1;0 | Phosphatidylinositol (19:1_18:1) |  |
| PI 20:0;0_18:1;0 | Phosphatidylinositol (20:0_18:1) |  |
| PI 20:1;0_18:0;0 | Phosphatidylinositol (20:1_18:0) |  |
| PI 20:0;0_18:2;0 | Phosphatidylinositol (20:0_18:2) |  |
| PI 20:1;0_18:1;0 | Phosphatidylinositol (20:1_18:1) |  |
| PI 22:1;0_16:1;0 | Phosphatidylinositol (22:1_16:1) |  |
| PI 20:1;0_18:2;0 | Phosphatidylinositol (20:1_18:2) |  |
| PI 20:2;0_18:1;0 | Phosphatidylinositol (20:2_18:1) |  |
| PS 14:0;0_17:1;0 | Phosphatidylserine (14:0_17:1) |  |
| PS 15:0;0_16:1;0 | Phosphatidylserine (15:0_16:1) | SLM:000077707 |
| PS 14:0;0_18:1;0 | Phosphatidylserine (14:0_18:1) | SLM:000077600 |
| PS 14:1;0_18:0;0 | Phosphatidylserine (14:1_18:0) | SLM:000077655 |
| PS 15:0;0_17:1;0 | Phosphatidylserine (15:0_17:1) |  |
| PS 16:1;0_16:0;0 | Phosphatidylserine (16:1_16:0) |  |
| PS 14:0;0_18:2;0 | Phosphatidylserine (14:0_18:2) | SLM:000077601 |
| PS 16:1;0_16:1;0 | Phosphatidylserine (16:1_16:1) | SLM:000077814 |
| PS 15:0;0_18:1;0 | Phosphatidylserine (15:0_18:1) | SLM:000077711 |
| PS 16:0;0_17:1;0 | Phosphatidylserine (16:0_17:1) |  |
| PS 15:0;0_18:2;0 | Phosphatidylserine (15:0_18:2) | SLM:000077712 |
| PS 15:1;0_18:1;0 | Phosphatidylserine (15:1_18:1) |  |
| PS 16:1;0_17:1;0 | Phosphatidylserine (16:1_17:1) |  |
| PS 16:0;0_18:1;0 | Phosphatidylserine (16:0_18:1) | SLM:000077765 |
| PS 16:1;0_18:0;0 | Phosphatidylserine (16:1_18:0) | SLM:000077817 |
| PS 16:0;0_18:2;0 | Phosphatidylserine (16:0_18:2) | SLM:000077766 |
| PS 16:1;0_18:1;0 | Phosphatidylserine (16:1_18:1) | SLM:000077818 |
| PS 17:1;0_17:1;0 | Phosphatidylserine (17:1_17:1) |  |
| PS 16:0;0_18:3;0 | Phosphatidylserine (16:0_18:3) | SLM:000077767 |
| PS 16:1;0_18:2;0 | Phosphatidylserine (16:1_18:2) | SLM:000077819 |
| PS 16:2;0_18:1;0 | Phosphatidylserine (16:2_18:1) | SLM:000077870 |
| PS 17:0;0_18:1;0 | Phosphatidylserine (17:0_18:1) | SLM:000077921 |
| PS 17:1;0_18:0;0 | Phosphatidylserine (17:1_18:0) |  |
| PS 17:0;0_18:2;0 | Phosphatidylserine (17:0_18:2) | SLM:000077922 |
| PS 17:1;0_18:1;0 | Phosphatidylserine (17:1_18:1) |  |
| PS 18:1;0_18:1;0 | Phosphatidylserine (18:1_18:1) | SLM:000078020 |
| PS 18:2;0_18:0;0 | Phosphatidylserine (18:2_18:0) |  |
| PS 18:2;0_18:1;0 | Phosphatidylserine (18:2_18:1) |  |
| PS 18:2;0_18:2;0 | Phosphatidylserine (18:2_18:2) | SLM:000078069 |
| PS 18:3;0_18:1;0 | Phosphatidylserine (18:3_18:1) |  |
| ST 28:3;0 |  |  |
| TAG 36:0;0 | Triacylglycerol (36:0) | SLM:000308209 |
| TAG 41:0;0 | Triacylglycerol (41:0) | SLM:000308218 |
| TAG 42:0;0 | Triacylglycerol (42:0) | SLM:000308221 |
| TAG 42:1;0 | Triacylglycerol (42:1) | SLM:000308222 |
| TAG 43:0;0 | Triacylglycerol (43:0) | SLM:000308226 |
| TAG 44:0;0 | Triacylglycerol (44:0) | SLM:000308231 |
| TAG 44:1;0 | Triacylglycerol (44:1) | SLM:000308232 |
| TAG 45:0;0 | Triacylglycerol (45:0) | SLM:000308237 |
| TAG 46:0;0 | Triacylglycerol (46:0) | SLM:000308243 |
| TAG 46:1;0 | Triacylglycerol (46:1) | SLM:000308244 |
| TAG 46:2;0 | Triacylglycerol (46:2) | SLM:000308245 |
| TAG 47:0;0 | Triacylglycerol (47:0) | SLM:000308250 |
| TAG 47:1;0 | Triacylglycerol (47:1) | SLM:000308251 |
| TAG 47:2;0 | Triacylglycerol (47:2) | SLM:000308252 |
| TAG 48:0;0 | Triacylglycerol (48:0) | SLM:000308257 |
| TAG 48:1;0 | Triacylglycerol (48:1) | SLM:000308258 |
| TAG 48:2;0 | Triacylglycerol (48:2) | SLM:000308259 |
| TAG 48:3;0 | Triacylglycerol (48:3) | SLM:000308260 |
| TAG 49:1;0 | Triacylglycerol (49:1) | SLM:000308267 |
| TAG 49:2;0 | Triacylglycerol (49:2) | SLM:000308268 |
| TAG 49:3;0 | Triacylglycerol (49:3) | SLM:000308269 |
| TAG 50:1;0 | Triacylglycerol (50:1) | SLM:000308276 |
| TAG 50:2;0 | Triacylglycerol (50:2) | SLM:000308277 |
| TAG 50:3;0 | Triacylglycerol (50:3) | SLM:000308278 |
| TAG 50:4;0 | Triacylglycerol (50:4) | SLM:000308279 |
| TAG 50:5;0 | Triacylglycerol (50:5) | SLM:000308280 |
| TAG 51:2;0 | Triacylglycerol (51:2) | SLM:000308287 |
| TAG 51:3;0 | Triacylglycerol (51:3) | SLM:000308288 |
| TAG 51:4;0 | Triacylglycerol (51:4) | SLM:000308289 |
| TAG 52:2;0 | Triacylglycerol (52:2) | SLM:000308298 |
| TAG 52:3;0 | Triacylglycerol (52:3) | SLM:000308299 |
| TAG 52:4;0 | Triacylglycerol (52:4) | SLM:000308300 |
| TAG 52:5;0 | Triacylglycerol (52:5) | SLM:000308301 |
| TAG 53:3;0 | Triacylglycerol (53:3) | SLM:000308310 |
| TAG 53:4;0 | Triacylglycerol (53:4) | SLM:000308311 |
| TAG 53:5;0 | Triacylglycerol (53:5) | SLM:000308312 |
| TAG 54:3;0 | Triacylglycerol (54:3) | SLM:000308323 |
| TAG 54:4;0 | Triacylglycerol (54:4) | SLM:000308324 |
| TAG 54:5;0 | Triacylglycerol (54:5) | SLM:000308325 |
| TAG 54:6;0 | Triacylglycerol (54:6) | SLM:000308326 |
| TAG 56:3;0 | Triacylglycerol (56:3) | SLM:000308349 |
| TAG 56:4;0 | Triacylglycerol (56:4) | SLM:000308350 |
| TAG 58:3;0 | Triacylglycerol (58:3) | SLM:000308377 |
